# Novel Quinazolin-2,4-Dione Hybrid Molecules as Possible Inhibitors Against Malaria: Synthesis and *in silico* Molecular Docking Studies

**DOI:** 10.3389/fmolb.2020.00105

**Published:** 2020-06-05

**Authors:** Aboubakr Haredi Abdelmonsef, Mahmoud Eldeeb Mohamed, Mohamed El-Naggar, Hussain Temairk, Ahmed Mohamed Mosallam

**Affiliations:** ^1^Chemistry Department, Faculty of Science, South Valley University, Qena, Egypt; ^2^Chemistry Department, Faculty of Sciences, University of Sharjah, Sharjah, United Arab Emirates

**Keywords:** malaria, *pf*DHODH, hybrid molecules, quinazolin-2,4-dione, N-heterocyclic moieties, docking study

## Abstract

The research explores the synthesis of a series of novel hybrid quinazolin-2,4-dione analogs bearing acetyl/amide bridged-nitrogen heterocyclic moieties such as azetidinone, pyrrole, oxazole, oxadiazole, thiazole, pyrazole, and thiazolidine scaffolds **2-16**. The newly synthesized compounds were structurally confirmed by means of IR, ^1^H-NMR, ^13^C-NMR, MS and elemental analysis. In addition, an *in silico* molecular docking analysis of new compounds and standard drug (Chloroquine) has been performed to analyze the binding modes of interaction to the putative active site of *Plasmodium falciparum* Dihydroorotate dehydrogenase (*pf*DHODH). Aiming to search for potentially better antimalarials, a modern approach has been undertaken to identify new quinazolin-2,4-dione derivatives targeting *pf*DHODH. The identification of antimalarial activity of the newly synthesized compounds by using experimental techniques is expensive and requires extensive pains and labor. The compound **11** showed the highest binding affinity against *pf*DHODH. Moreover, the electrostatic potential (ESP) of the docked molecules was also calculated. Further, the pharmacokinetic properties (ADMET) of the prepared compounds were predicted through *in silico* technique.

**Graphical Abstract:**
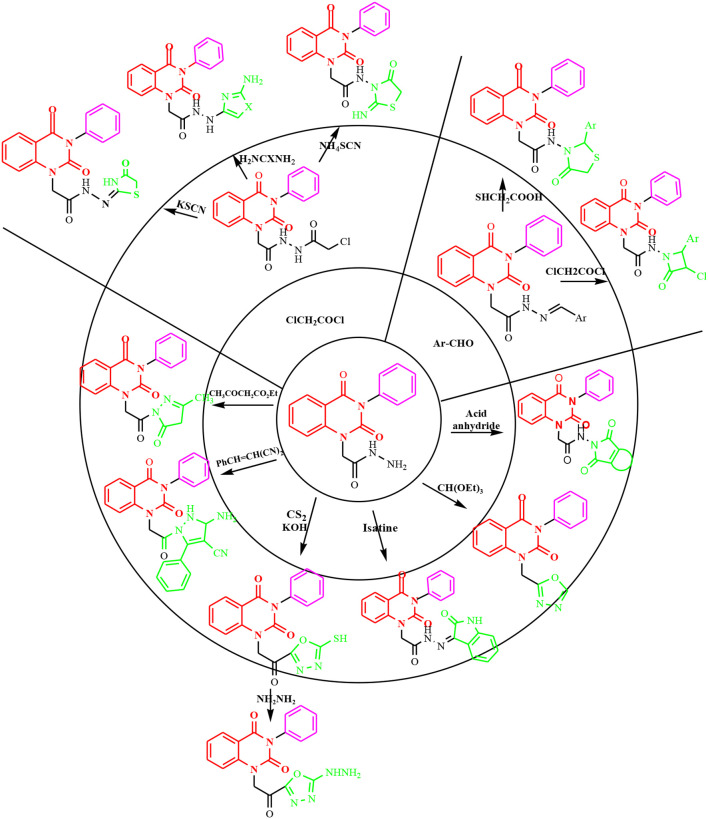
Synthesis of new quinazolin-2,4-dione hybrid molecules.

## Introduction

Malaria is a parasitic disease caused by single-celled microorganisms of *Plasmodium* group (Nivsarkar et al., 2005; Sahu et al., 2019). It is a widespread health problem in the tropical regions (Campbell, 1997; Autino et al., 2012). According to WHO, about 219 million cases and 430,000 related deaths worldwide of malaria were reported in the year 2017 (Talapko et al., 2019). *Plasmodium falciparum* Dihydroorotate dehydrogenase is an essential enzyme which is predominantly responsible for the growth of malaria parasite (Talapko et al., 2019; Vikram and Mishra, 2019). These parasites are transmitted to the human by the bites of infected female anopheles mosquitoes. Four species of plasmodia are commonly known to cause human malaria: *P. falciparum, P. vivax, P. ovale*, and *P. malariae* (Oguike et al., 2011). The 85% of malarial deaths are caused by the intracellular protozoan parasite *Plasmodium falciparum* (Blasco et al., 2017). Therefore, the targeting of the human enzyme *pf*DHODH is the main concern in combating malaria disease.

Molecular hybridization concept has gained much interest in last decades (Claudio et al., 2007). The synthesis of hybrid molecules by combination of two different biologically moieties gave promising information for medicinal chemistry, as they exhibited anti-fungal, anti-malarial, anti-cancer, and anti- inflammatory activities (Gómez and Kouznetsov, 2013). Quinazolin-2,4-diones are such interesting structural cores that found to have significant attention in medicinal and pharmaceutical chemistry (Hassan et al., 2013a,b; El-Sheshtawy et al., 2017; Abdelmonsef and Mosallam, 2020; El-Naggar et al., 2020). Heterocycles bearing nitrogen such as azetidinone, pyrrole, oxazole, oxadiazole, thiazole, pyrazole, and thiazolidine scaffolds are reported to possess a broad spectrum of applications in medicinal, biological as well as pharmaceutical activities as anti-fungal, anti-bacterial, anti-tumor, and anti-inflammatory drugs (Tanitame et al., 2004; Dua et al., 2011; Sokolova et al., 2017). Nowadays, the synthesis of nitrogen containing heterocyclic systems attached to quinazolin-2,4-dione ring have drawn considerable importance owing to their enormous applications in medicinal and pharmaceutical chemistry (Patel et al., 2018).

Guided by these findings, and as a part of our efforts to synthesis of novel dual hybrid molecules, our group planned to incorporate the quinazolin-2,4-dione moiety as a main scaffold attached with various N-heterocyclic moieties at nitrogen N-1 position through acetyl/amide linkage **2-16** which can be used as possible antimalarials targeting *pf*DHODH using structure-based drug discovery approach. Chloroquine is an aminoquinoline which belongs to a class of potent anti-malarial drugs (Bawa et al., 2010) and has been selected as a reference ligand in order to compare its binding affinity and their of the prepared compounds toward *pf*DHODH. The electrostatic potential of the molecules was performed through the determination of the electron charge density, to understand the intermolecular interactions to the target enzyme (Drissi et al., 2015). In addition, the pharmacological and pharmacokinetics properties of these compounds were also further calculated using *in silico* methods such as Mol inspiration and admetSAR web servers.

## Results and Discussion

### Chemistry

**Scheme 1:**
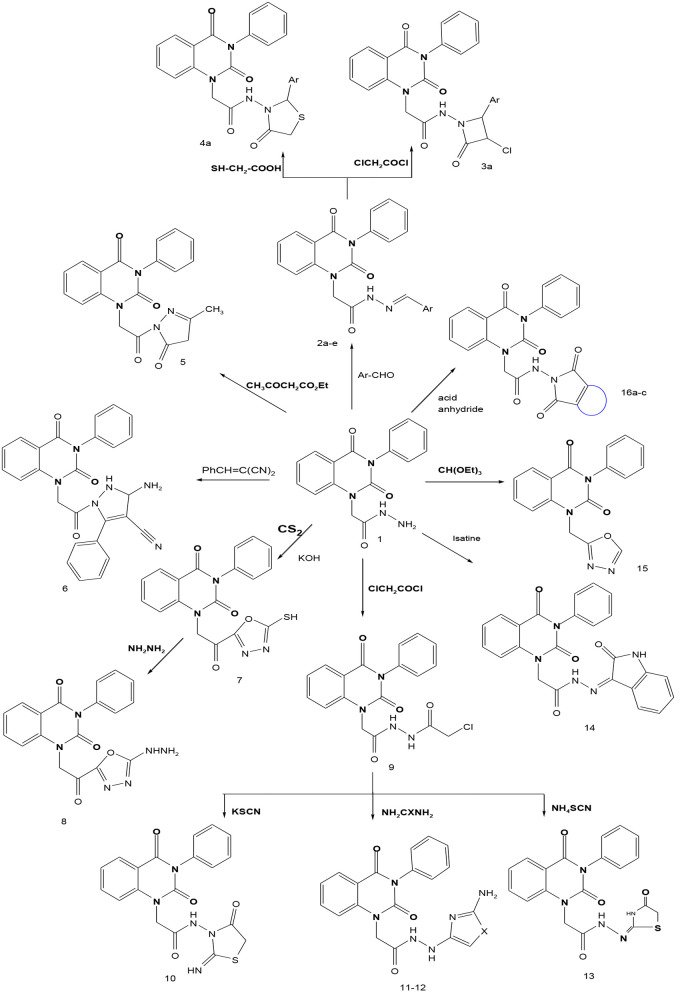
Synthesis of quinazolin-2,4-dione analog bearing N-heterocyclic moieties **2-16**. ArCHO (a- benzaldehyde, b- 4-nitrobenzaldehyde, c- 4-chloro benzaldehyde, d- salicylaldehyde, and e- thiophene-2-carboxaldehyde). X=O, and S. Acid anhydrides: a- maleic, b- phthalic, and c- tetrachlorophthalic anhydride.

**Scheme 2:**
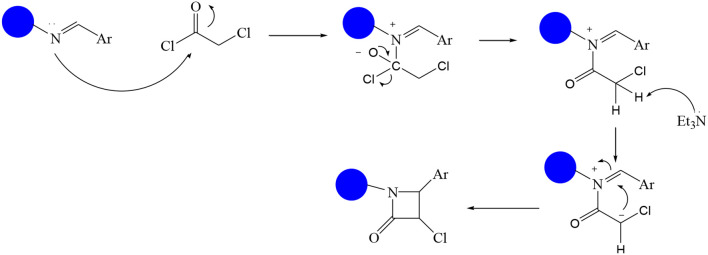
Synthesis of azetidinone derivative **3a**.

**Scheme 3:**
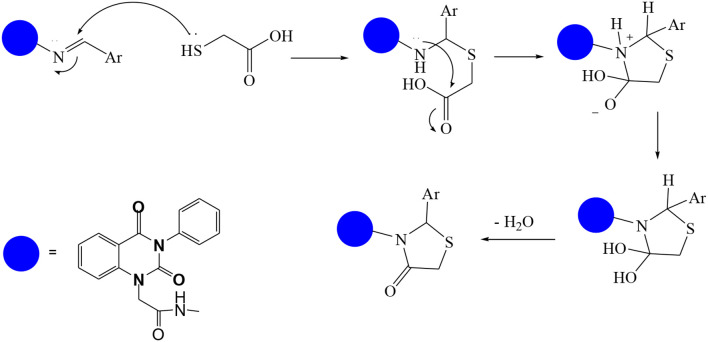
Reaction protocol for the synthesis of compound **4a**.

**Scheme 4:**
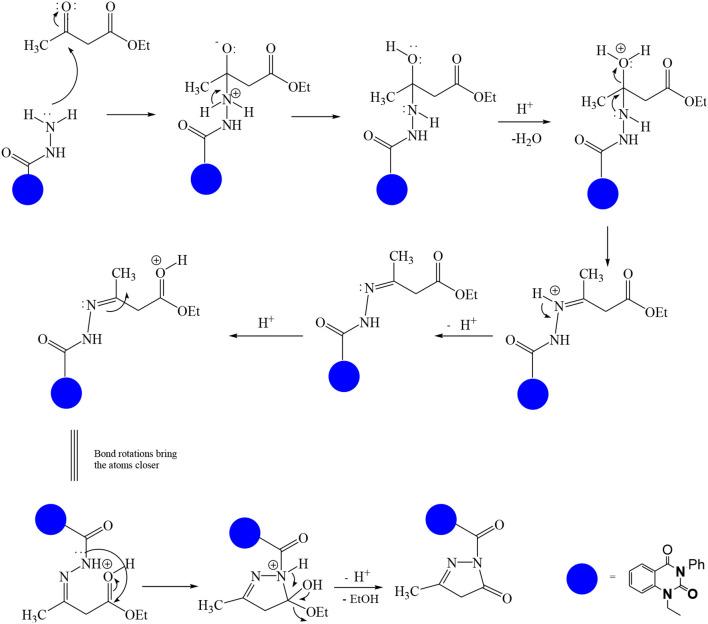
Mechanism of formation of compound **5**.

**Scheme 5:**
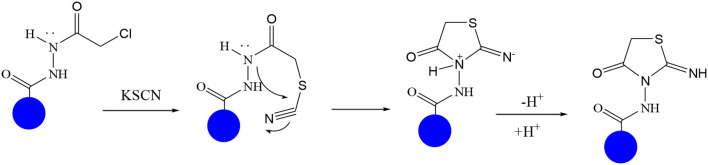
Synthetic strategy of analog **10**.

**Scheme 6:**
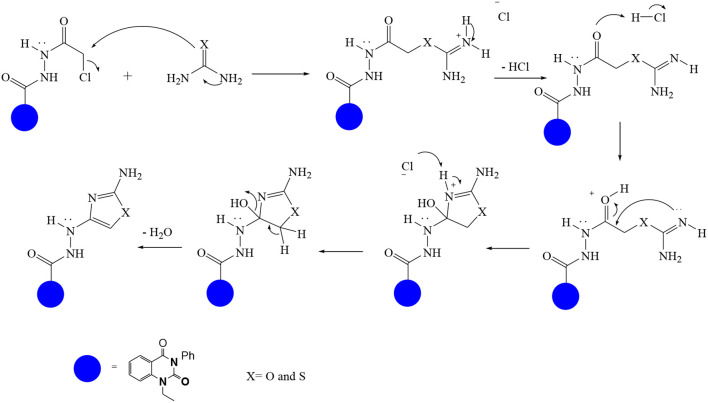
Plausible mechanism for the synthesis of compounds **11-12**.

In continuation of our efforts, herein we reported the synthesis of 15 structural hybrid molecules of quinazolindione coupled to four and five membered N-heterocycles at N-1 position by using the synthetic protocols as depicted in Scheme 1. The acetyl/amide group serves as a connecting bridge fragment between both nucleolus. The carbohydrazide analog **1**, was obtained using the method described by our group (El-Naggar et al., 2020) *via* the treatment of 2,4-Dioxo-3-phenyl-3,4-dihydroquinazolin-1(2H)-yl) acetate with hydrazine hydrate. The compound **1** is used as a reaction intermediate in construction of four and five membered heterocyclic systems such as azetidine, pyrrole, oxazole, oxadiazole, thiazole, and thiazolidine moieties. A series of Schiff bases **2a-e** were synthesized by reaction of so formed 2-(2,4-dioxo-3-phenyl-3,4-dihydroquinazolin-1(2H)-yl)acetohydrazide **1** with an equimolar amount of different aromatic aldehydes namely, benzaldehyde, 4-nitrobenzaldehyde, 4-chlorobenzaldehyde, salicylaldehyde, and thiophene-2-carboxaldehyde, respectively. IR spectra of benzylidine hydrazide derivatives **2a-e** provide valuable information regarding nature of functional group present in these compounds. The bands around 1,576–1,625 cm^−1^ suggested the presence of CH=N (azomethine linkage) in the compounds. In addition, the ^1^H-NMR spectra for compounds **2a-e** revealed singlet signal at δ 4.37 ppm characteristic for N-CH_2_-CO, multiplet signals at the range of δ 7.25–8.06 ppm for aromatic protons, in addition, the important signal at δ 5.81 ppm equivalent to one hydrogen due to the CH=N group of the Schiff bases, plus singlet signals at 11.51 ppm assigned for NH's, respectively, which were exchangeable by D_2_O. The so formed Schiff base **2a** was converted to azetidin-2-one **3a** by [2+2] cycloaddition reaction with chloroacetyl chloride as shown in Scheme 2. The desired compound **3a** has been approved by disappearance of absorption band of 1,576 cm^−1^ which is related to azomethine group (-N=CH), and appearance of the carbonyl azetidinone band at 1,660 cm^−1^. Furthermore, the appearance of new band at 673 cm^−1^ due to the stretching for C-Cl bond. Moreover, it can be confirmed on the basis of ^1^H-NMR, by disappearance of azomethine signal at δ 5.81 ppm and the appearance of two signals in the region δ 5.48–6.45 ppm due to aliphatic protons of new azetidinone ring, -N-CH-Ar and –CO-CH-Cl. Heterocyclization of compound **2a** with thioglycolic acid yielded quinazolin-2,4-dione analog attached to 2-thiazolidine **4a**, by nucleophilic addition to the carbonyl group of thioglycolic acid. The structure of the resulted compound **4a** was efficiently characterized by IR and ^1^H-NMR. For instance, the FT-IR spectrum showed the appearance of stretching frequency of amidic carbonyl group (N-C=O) at 1,726 cm^−1^ due to the thiazolinone ring and this considered the most characteristic evidence for the success of cyclization step. Beside the disappearance of –N=C group at 1,576 cm^−1^ for Schiff base **2a**. In addition, ^1^H-NMR spectrum of compound **4a** exhibited new characteristic signal for CH_2_ group adjacent to S (CH_2_-S) appeared at δ 4.37 ppm; this again confirmed the formation of thiazolinone ring. The proposed mechanism of this reaction is declared in Scheme 3. Compound **5** was resulted from reaction of carbohydrazide analog **1** with β-ketoester like ethyl acetoacetate through Knorr pyrazole synthesis as shown in Scheme 4 (Knorr, 1883). The latter compound showed in its ^1^H-NMR spectrum characteristic peaks at δ 1.36, 4.38, and 7.25–8.07 ppm attributed to CH_3_, CH_2_ and aromatic protons, respectively. Moreover, 3-Amino-1-[2-(2,4-dioxo-3-phenyl-3,4-dihydro-2H-quinazolin-1-yl)-acetyl]-5-phenyl-2,3-dihydro-1H-pyrazole-4-carbonitrile **6** was obtained employing the reaction of compound **1** with benzylidene malononitrile. The structure of compound **6** was corroborated by its IR, ^1^H-NMR and elemental analysis. The IR spectra of the resulted **6** showed absorption bands at 3,400 and 2,225 cm^−1^ for NH_2_ and C=N groups, respectively. In addition, the formation of compound **8** was prepared accordingly, by heating of carbohydrazide **1** with CS_2_ in presence of alc. KOH to yield compound **7**, and then the latter compound was separated and treated with hydrazine hydrate. The ^1^H-NMR spectroscopic technique used to characterize the newly synthesized compound **8**; it showed new characteristic signals for NH and NH_2_ groups at 11.53 and 8.73 ppm, respectively. Chloro-acetic acid N'-[2-(2,4-dioxo-3-phenyl-3,4-dihydro-2H-quinazolin-1-yl)-acetyl]-hydrazide **9**, which was synthesized by reaction of compound **1** with chloroacetyl chloride (El-Naggar et al., 2020), is used as starting material to synthesize of compounds **10-13**. The reaction of compound **9** with potassium thiocyanate through Dimroth type rearrangement to yield the annealed compound **10** as declared in Scheme 5. The FT-IR spectrum of the compound **10** showed the appearance of stretching band of C=NH group at 1,580 cm^−1^. On the other hand, the compound **9** underwent heterocyclization with urea, and thiourea to afford compounds **11** and **12**, respectively, as declared in Scheme 6. The ^1^H-NMR spectra of both compounds showed new characteristic signal at 8.72 ppm for NH_2_ group, in addition, appearance of the CH proton in interference with the aromatic protons in the same range for the newly synthesized compounds, which is considered the most characteristic evidence for the success of cyclization step and formation of oxazole and thiazole rings, respectively. Preparation of compound **13** was achieved by heterocyclization of compound **9** with ammonium thiocyanate. ^1^H-NMR spectrum of compound **13** shows new signal for CH_2_ group at 4.3 ppm of new thiazolinone ring. The formation compounds **14** and **15** were attempted through refluxing of carbohydrazide **1** with Isatine and Triethyl orthoformate, respectively. The ^1^H-NMR spectrum of compound **14** shows new signal for NH group at 8.74 ppm of new Isatine ring introduced to hydrazide **1**. The ^1^H-NMR spectrum of compound **15** showed disappearance of signals related to NH and NH_2_ groups, and appearance of new signal related to CH proton of new oxadiazole ring, which is in interference with the aromatic protons. The FT-IR spectrum of the resulted compound showed the appearance of –N=C group at 1,600 cm^−1^ for oxadiazole derivative **15**. Finally, reaction of carbohydrazide analog **1** with various acid anhydrides like maleic, phthalic, and tetrachlorophthalic anhydride through dehydrative condensation reaction, gives the corresponding imides **16a-c**, respectively. The ^1^H-NMR/IR spectra of the resulted imides showed disappearance of signal/band related to NH_2_ group, with appearance of new signals/bands. The mass spectra of compounds **2-16** add additional confirmation for elucidation of the structures which showed molecular ion peaks corresponding to molecular formula of these compounds further confirmed the assigned structures. CHN analysis was confirmed the validity of the assumed structures of the newly synthesized compounds (see Experimental section).

### *In silico* study

**Figure 1:**
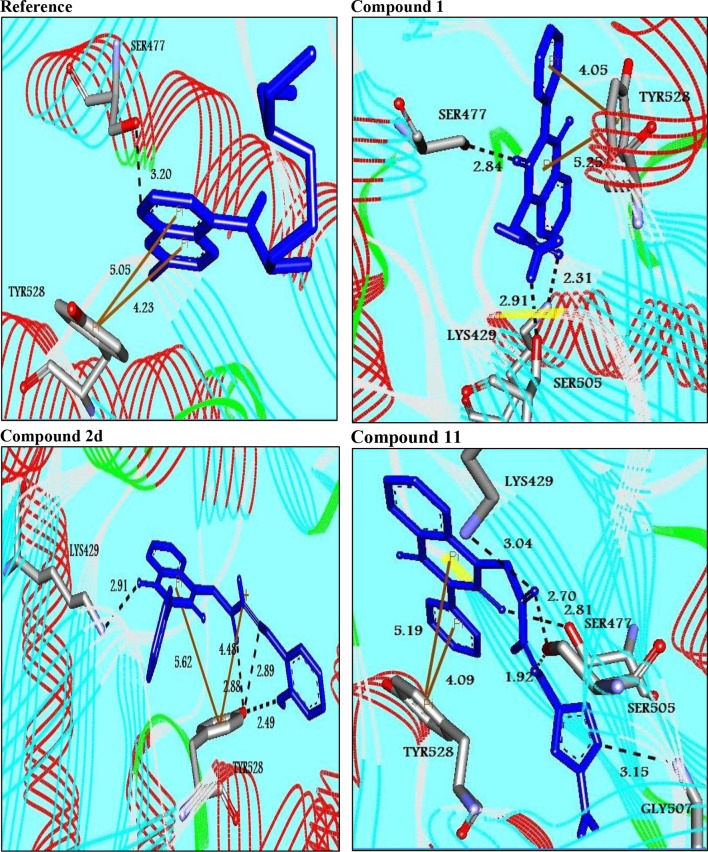
Molecular interactions between the best binders and *pf*DHODH. Three-dimensional of docking poses of the reference and three best binding compounds in the active pocket of *pf*DHODH. Blue stick models represented the docked compounds, and gray models represented the active site region. H-bond interactions are shown in black dotted lines. π- interactions are shown in orange lines.

**Figure 2:**
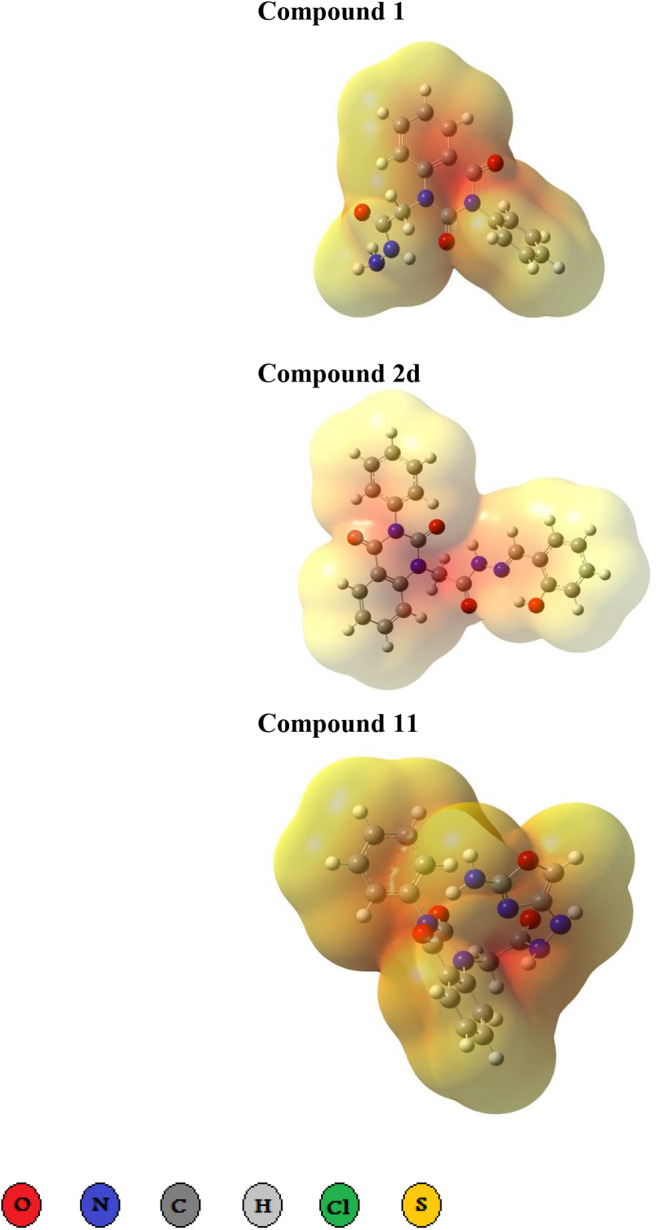
Three dimensional representation of electrostatic potential around the best three docked molecules. Electrostatic potential maps around the best binders **1, 2d**, and **11**. The red region represents highly electron density charges (electronegative), while pale yellow represents electropositive part.

Three- dimensional crystal structure of *pf*DHODH (PDB ID: 1tv5) was retrieved from RCSB Protein Data Bank web server (Hurt et al., 2006) (Figure S1). The PDB file of the target enzyme was optimized by energy minimization and prepared prior to docking by removal of water molecules and atomic clashes. Moreover, H-atoms and gasteiger charges were added to the target enzyme for correct ionization and tautomeric states of residues. The protonation states of ionizable groups in receptor were theoretically calculated according to Propka (Table S1). In accordance with previously literatures, the putative binding site residues of the target enzyme *pf*DHODH are PHE171, LEU172, CYS175, GLY181, CYS184, HIS185, PHE188, LEU189, PHE227, ILE263, ARG265, TYR528, LEU531, VAL532, GLY535, and MET536 (Vikram and Mishra, 2019), which found to be playing a catalytic role by forming the intermolecular interactions with the ligand molecules. Grid was then allocated around the region of interest (binding site) to perform further structure-based screening approach (Abdelmonsef et al., 2016; Aboubakr et al., 2016). In-house set of 22 analogs of quinazolindione derivatives was used for study. In addition, ligand molecules must be prepared to create 3D geometries, assign proper bond orders, and generate accessible tautomerization and ionization sates prior for molecular docking studies (Figure S2). A library of 22 compounds was subjected to PyRx-Virtual screening tool version 8.0 through inbuilt Autodock 4.2 with the Lamarckian genetic algorithm (LGA) as scoring function (Morris et al., 1639), in order to carry the docking simulation with *pf*DHODH. Docking approach was performed on default parameters of number of generation and energy evaluation for 10 steps of run. Molecular docking study was accomplished to estimate the binding mode interactions between a novel series of prepared molecules and active site region of the target enzyme (Dasari et al., 2017; Rondla et al., 2017; Shehadi et al., 2020). Upon the completion of each docking approach, a maximum output of 9 distinct conformational clusters was calculated for each ligand. The molecule with lowest binding energy (i.e., more negative) indicates highest binding affinity to enzyme (Hussein et al., 2018; Abdelmonsef, 2019). The predicted binding energies were computed in units of kcal/mol. Generally, the target compounds fit well into the binding regions of *pf*DHODH with different binding energies. Docking of the target analogs to *pf*DHODH yielded scores between −12.2 and −5.7 kcal/mol, compared to −7.2 kcal/mol for reference drug (Chloroquine), and disclosed the derivative **11** as a lead compound. On close inspection of Table 1, Figure 1, and (Figure S3) potential interactions (hydrogen bonds, π-π stacking, π-cation, and π-sigma), bond lengths and atoms involved in these interactions were observed between the synthesized molecules and reference with enzyme. The analog **1** was successfully docked into *pf*DHODH with binding energy −9.6 kcal/mol. It exhibited hydrogen bond and π-π interactions (π-stacking) with the binding regions via four amino acid residues namely LYS429, SER477, SER505, and TYR528 at the distance of 2.91, 2.84, 2.31, 5.25, and 4.05 Å, respectively. In case of compounds **2a-e**; all of them exhibited H-bond interactions, in addition they showed π-cation interactions except **2e** with ARG544, ARG253, TYR207, and TYR528 at the distances of 6.47, 4.66, 5.93, 4.70, and 4.48 Å, respectively. Furthermore, they exhibited π-π interactions (edge to face) with TYR207 and TYR528, respectively, except derivatives **2b** and **2e**. Introduction of electron donating group (OH) on phenyl ring causing high activity as shown in compound **2d** (best binding energy of them = −10.5 kcal/mol) (Metwally et al., 2018). On the other hand, the derivatives **2b** (−7.1 kcal/mol) and **2c** (−8.0 kcal/mol) with electron withdrawing groups such as –NO_2_ (strong) and –Cl (weak), respectively, exhibited less binding affinities in comparison to derivative **2d**. Introducing of electron withdrawing groups (–NO_2_ and –Cl) on phenyl ring causing less activity of the compounds (Metwally et al., 2018). The compound **3a** exhibited H-bond interactions with HIS185, ASN274, LYS429, SER477, and TYR528 at the distances of 2.95, 2.61, 2.96, 2.42, 2.97, and 2.95 Å, respectively. In addition to π-cation and π-sigma interactions with LYS429 and ILE263, respectively. The analog **4a** formed π-π (edge to face, and face to face), and π-cation interactions with the residues ASN547, TYR207, and ARG544 at 2.93, 2.96, 5.12, 5.70 (edge to face), 3.70 Å (face to face), respectively. Compound **5** showed H-bond interactions with LYS260 and ARG262 at the distances of 3.12 and 2.92 Å. The product **6** of −8.5 kcal/mol exhibited three types of interactions like H-bond, π-π, and π-cation interactions with ASN547, TYR207, and ARG544 at 2.95, 4.28, 5.06, and 5.82 Å, respectively. In comparison between compounds **7** (−7.5 kcal/mol) and **8** (−8.4 kcal/mol); both of aromatic thiol and aromatic amine are electron donating groups, herein we noted that the presence of amino group enhanced the binding energy of compound **8** than compound **7** which contains thiol group. Compound **9** exhibited H-bond, π-π, π-cation interactions with the residues GLY507, SER529, TYR528, LYS229, and LYS429 at 3.01, 2.95, 3.69, 3.88, and 5.98 Å, respectively. Compound **10** showed H-bond and π-π interactions with the residues SER205, ASN547, and TYR207 at 3.09, 2.88, and 5.06 Å, respectively. In addition, the best binding energy was reported by compound **11** (−12.2 kcal/mol) formed different H-bonds with LYS429, SER477, SER505, and GLYS507 and π-π stacking with TYR528. The analog **12** exhibited H-bond and π-cation interactions with TYR168, SER160, ASN195, and PHE165 at the distances of 2.97, 2.22, 2.13, 2.15, and 6.71 Å, respectively. While the compound **13** of −7.7 kcal/mol docked into *pf*DHODH through one H-bond and two π-π interactions with TYR555 and TYR207. Moreover, the analog **14** exhibited H-bond and π-cation interactions with the active site pocket of the target enzyme through ARG253, THR318. The product **15** formed three types of interactions H-bond, π-cation and π-π through ASN274, LYS429, and TYR528 at the distances of 2.95, 2.64, 2.80, 2.85, 5.52, and 4.60 Å, respectively. Finally, the imide **16a** formed H-bonds with SER477, 505, and 529, in addition to two π-π interactions with TYR528. In comparison between the phthalimides **16b-c**, we noted that the presence of four chlorine groups in compound **16c** caused the moderate binding energy (−8.6 kcal/mol) than compound **16b** (−8.8 kcal/mol) which contains un-substituted benzene ring. To further understand the nature of flexible docking, tyrosine TYR207 and 528 formed unique binding mode *via* π-π stacking with some docked molecules, because it contains a phenyl ring on its side-chain that is involved in forming these interactions with the compounds. On the other hand, arginine ARG253, 262, and 544 contain a positively charged guanidinium group H_2_N-C(=NH)-NH-, that is involved in forming π-cation interaction with compounds **2a, 2b, 2c, 4a, 6, 7**, and **14**. In addition, lysine LYS229, 429 also contain a positively charged amino on its side-chain (H_3_N^+^) that is involved in forming π-cation interactions with compounds **3a**, **8, 9**, and **15**. Meanwhile, the side chains of aromatic amino acids PHE165 and TYR528 in docked molecules **2d** and **12** provide a surface of negative electrostatic potential that can bind to a wide range of cations (π-cation interaction) through electrostatic interactions. These interactions may explain the highest potency toward *pf*DHODH enzyme (Zhang et al., 2015). Interestingly, the tested compounds (with quinazoline moiety) reported in this work and the reference compound (with quinoline moiety) are of similar moieties and are of important N-based heterocyclic aromatic compounds with a broad range of bioactivities (Kumar et al., 2010). The activities of the newly synthesized compounds could be due to heterocyclic core structures with acetyl/amide linkage. Moreover, the electrostatic potential of the docked molecules was also calculated to describe the intermolecular interactions to the target (Drissi et al., 2015). Figure 2 shows the theoretical maps in 3D dimensions of the electrostatic potential of the best three docked molecules. The obtained results could be used to explain the interaction between the molecules and the target enzyme. For example, the best binding compounds **1, 2d**, and **11** declared π-π stacking through their electronegative heterocyclic aromatic pyrimidine rings (Figure 2) and phenyl rings of TYR528. Further, an amidic carbonyl core -N-C=O of pyrimidine rings of the compounds exhibited three H-bonds with SER477 and LYS429, respectively. The electrostatic potential maps of the rest compounds are included as Figure S4. Moreover, the title analogs **1-16** were filtered depending on their Absorption, Distribution, Metabolism, Elimination and Toxicity (ADMET) (Table 2) and physicochemical significant descriptors (Table 3) using admetSAR and Mol inspiration online servers. The pharmacokinetic parameters (ADMET) reveal that newly synthesized compounds have better Human Intestinal Absorption (%HIA) score, and good Blood-Brain Barrier (BBB) values, which means that the compounds could be better absorbed by the human intestine. Furthermore, all prepared compounds displayed negative AMES toxicity and carcinogenicity test which means that the ligand molecules are non-mutagenic. In addition, the results of physiochemical properties predictions show interesting values for these compounds, whereas they have molecular weights in the range of 310–578 g/mol (<725), the partition coefficients of the compounds are <5. Also, the topological surface areas TPSA were found to be in the acceptable range (<140). In addition, the numbers of H-bond acceptors (HBA) and donors (HBD) in the tested compounds obey Lipinski's rule of five (Ro5), and were found to be in the range of 7–10 and 0–3, respectively. Finally, the synthesized compounds possess high numbers of rotatable bonds (3–6), which indicates that they are flexible. From all these results, we can conclude that all molecules exhibited good absorption, distribution and oral bioavailability within the body. These ligand molecules may be considered as potent drug candidates against *pf*DHODH and used as possible anti-malarial agents.

**Table 1:** Docking results and types of interactions between the title compounds 1–16 and active site region of *pf*DHODH enzyme.

	**Structure**	**Binding energy (kcal. mol^**−1**^)**	**Docked complex (amino acid–ligand) interactions**	**Distance (Å)**
Ref.	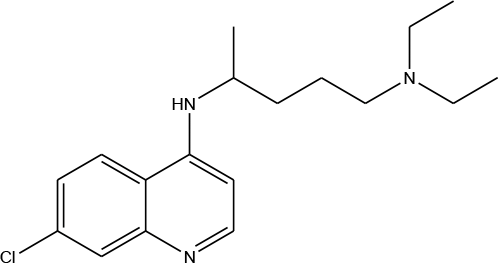	−7.2	**H-bonds** SER477:OG—reference **π**- **π** **interactions** TYR528—reference TYR528—reference	3.20 5.05 4.23
1	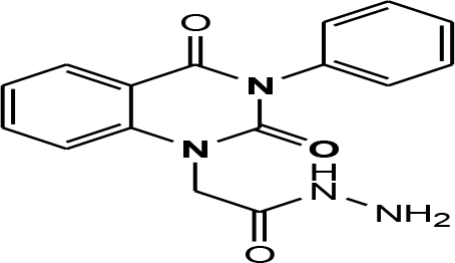	−9.6	**H-bonds** LYS429:NZ—compound 1 SER477:OG—compound 1 SER505:OG— compound 1 **π**- **π** **interactions** TYR528—compound 1 TYR528—compound 1	2.91 2.84 2.31 5.25 4.05
2a	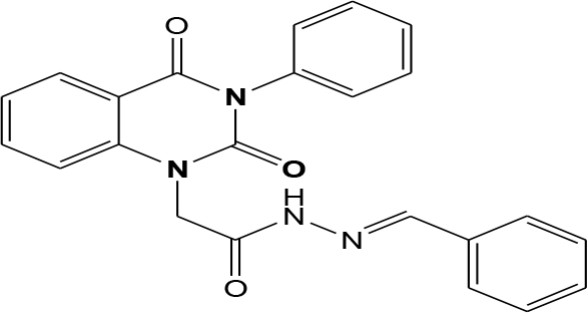	−7.7	**H-bonds** ASN547:ND2-compound 2a **π**- **π** **interactions** TYR207—compound 2a **π**- **cation interactions** ARG544:NH2–compound 2a	2.95 4.89 6.47
2b	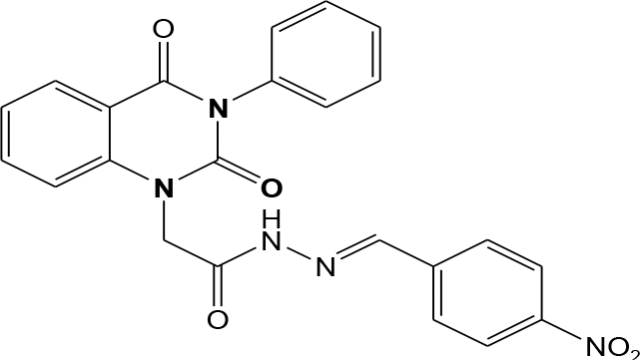	–7.1	**H-bonds** THR256:N—compound 2b THR256:OG1—compound 2b ASN315:ND2—compound 2b **π**- **cation interactions** ARG253:NH2—compound 2b	2.97 2.86 2.95 4.66
2c	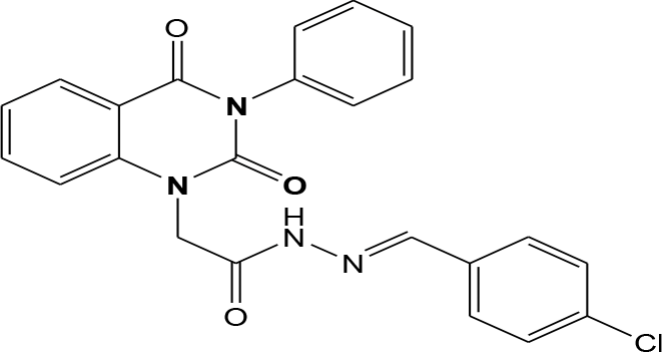	–8.0	**H-bonds** ARG544:NE—compound 2c ARG544:NH2—compound 2c **π**- **π** **interactions** TYR207—compound 2c **π**- **cation interactions** ARG544:NH1—compound 2c ARG544:NH2—compound 2c	2.87 2.95 4.85 5.93 4.70
2d	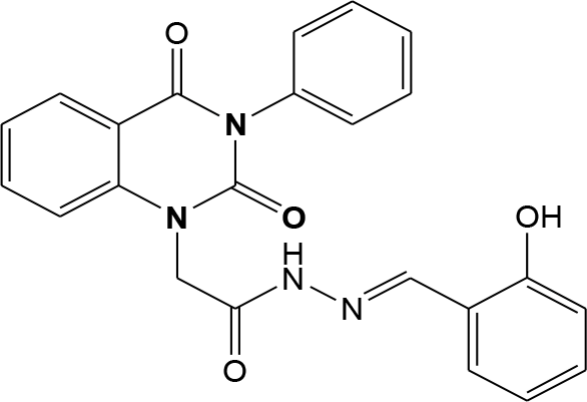	–10.5	**H-bonds** LYS429:NZ—compound 2d TYR528:OH—compound 2d TYR528:OH—compound 2d TYR528:OH—compound 2d **π**- **π** **interactions** TYR528—compound 2d **π**- **cation interactions** TYR528—compound 2d	2.91 2.88 2.89 2.49 5.62 4.48
2e	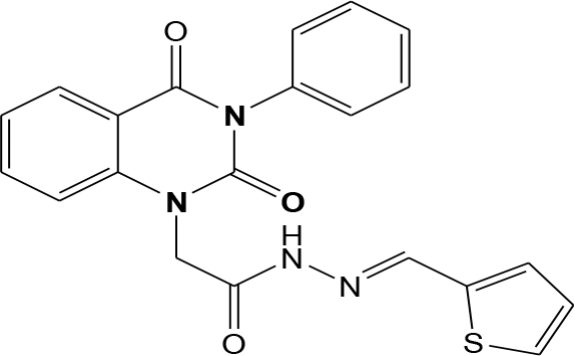	–7.5	**H-bonds** SER205:OG—compound 2e SER205:OG—compound 2e ASN547:ND2—compound 2e	2.96 2.93 2.82
3a	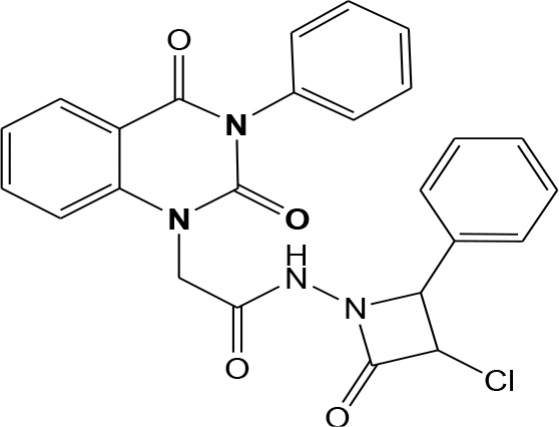	–5.7	**H-bonds** HIS185:ND1—compound 3a ASN274:ND2—compound 3a LYS429:NZ—compound 3a SER477:OG—compound 3a SER477:OG—compound 3a TYR528:OH—compound 3a **π**- **cation interactions** LYS429:NZ—compound 3a **π**- **sigma interactions** ILE263:CD1—compound 3a	2.95 2.61 2.96 2.42 2.97 2.95 5.84 3.46
4a	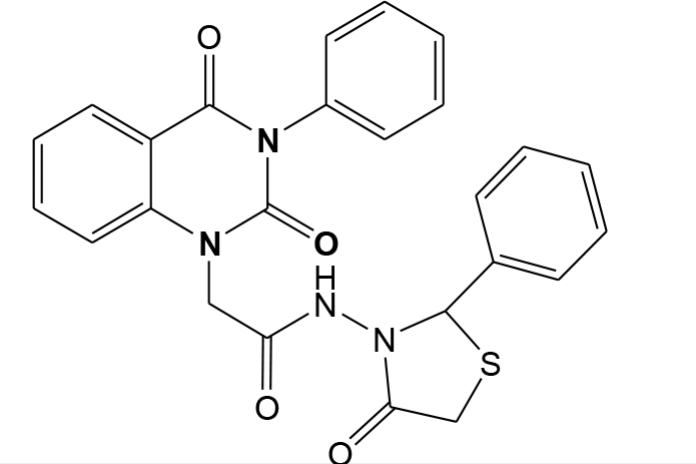	–8.3	**H-bonds** ASN547:ND2—compound 4a ASN547:ND2—compound 4a **π**- **π** **interactions** TYR207—compound 4a **π**- **cation interactions** ARG544:NH1—compound 4a ARG544:NH2—compound 4a	2.93 2.96 5.12 5.70 3.70
5	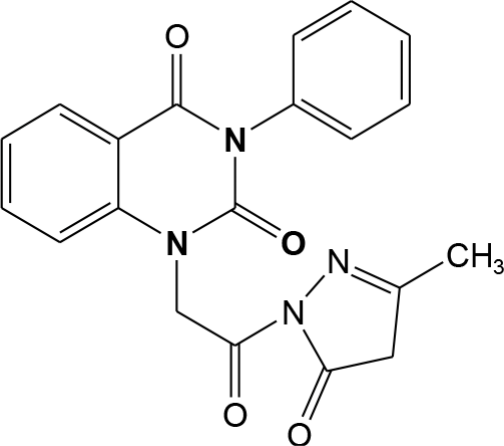	–8.2	**H-bonds** LYS260:NZ—compound 5 ARG262:NH2—compound 5	2.95 2.92
6	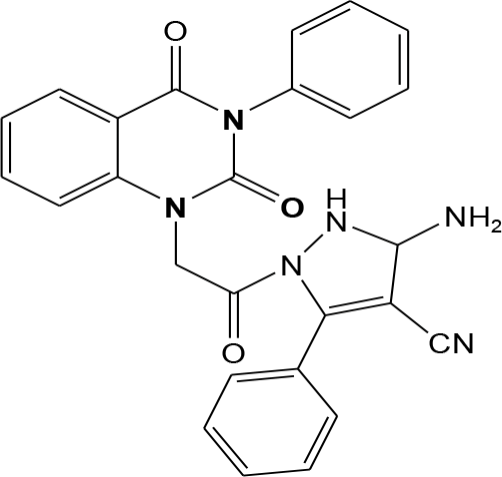	–8.5	**H-bonds** ASN547:ND2—compound 6 **π**- **π** **interactions** TYR207—compound 6 TYR207—compound 6 **π**- **cation interactions** ARG544:NH2—compound 6	2.95 4.28 5.06 5.82
7	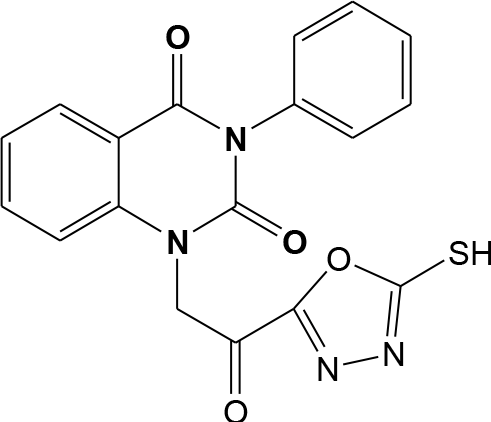	–7.5	**H-bonds** ASN230:ND2—compound 7 ASN279:ND2—compound 7 ASN279:ND2—compound 7 **π**- **cation interactions** ARG262:NH2—compound 7	2.95 2.93 2.96 5.24
8	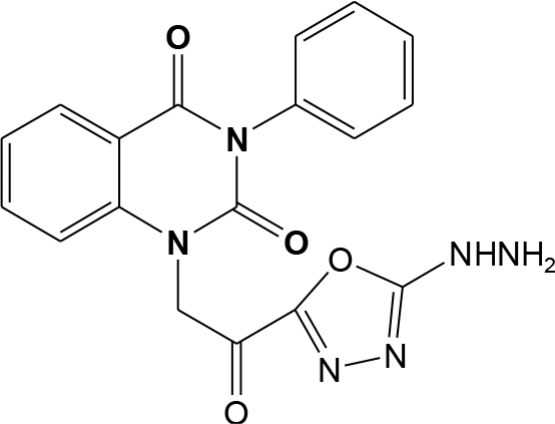	–8.4	**H-bonds** SER505:OG—compound 8 GLN526:O—compound 8 ILE508:O—compound 8 **π**- **π** **interactions** TYR528—compound 8 TYR528—compound 8 **π**- **cation interactions** LYS229:NZ—compound 8 LYS229:NZ—compound 8	2.96 2.37 2.43 4.40 3.62 4.17 5.71
9	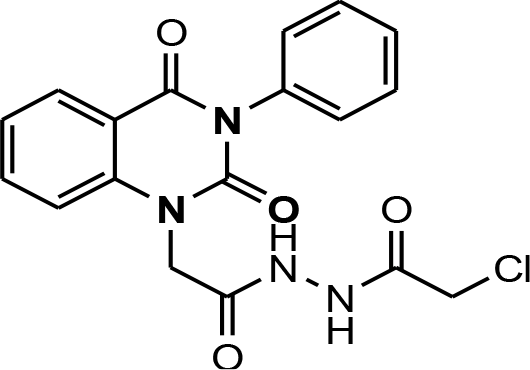	–9.5	**H-bonds** GLY507:N—compound 9 SER529:OG—compound 9 **π**- **π** **interactions** TYR528—compound 9 TYR528—compound 9 **π**- **cation interactions** LYS229:NZ—compound 9 LYS429:NZ—compound 9	3.01 2.95 3.69 3.69 3.88 5.98
10	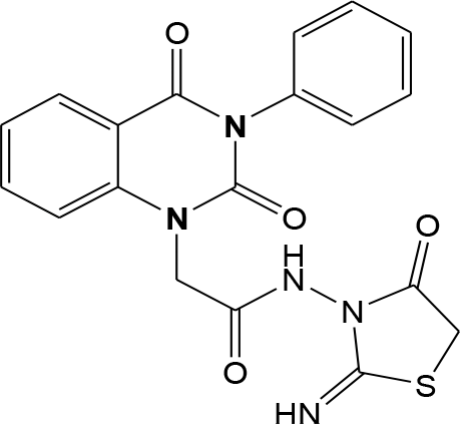	–7.8	**H-bonds** SER205:N—compound 10 ASN547:ND2—compound 10 **π**- **π** **interactions** TYR207—compound 10	3.09 2.88 5.06
11	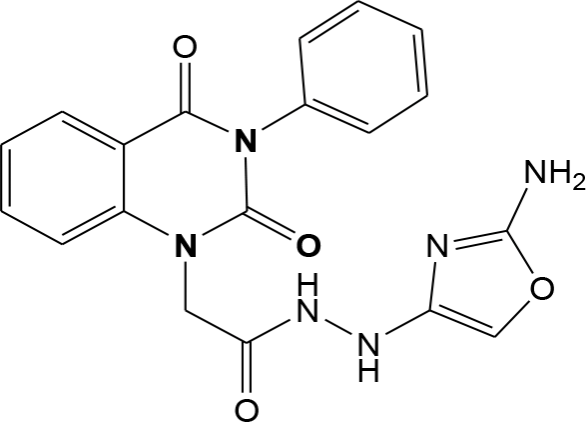	–12.2	**H-bonds** LYS429:NZ—compound 11 SER477:OG—compound 11 SER505:OG—compound 11 SER505:OG- compound 11 GLY507:N—compound 11 **π**- **π** **interactions** TYR528—compound 11 TYR528—compound 11	3.04 2.81 2.70 1.92 3.15 5.19 4.09
12	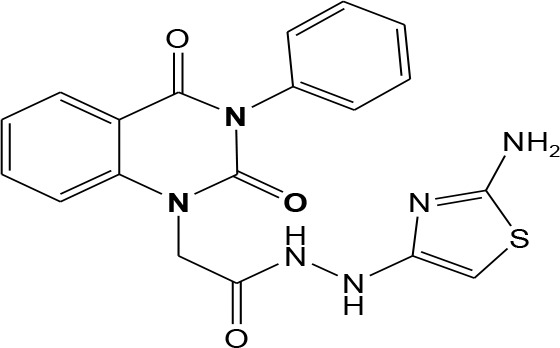	–7.6	**H-bonds** TYR168:OH—compound 12 SER160:O—compound 12 TYR168:OH—compound 12 ASN195:O—compound 12 **π**- **cation interactions** PHE165—compound 12	2.97 2.22 2.13 2.15 6.71
13	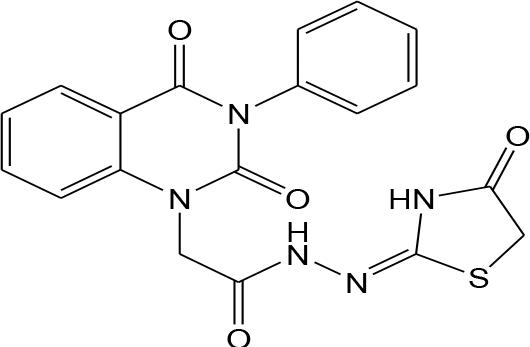	–7.7	**H-bonds** TYR555:O—compound 13 **π**- **π** **interactions** TYR207—compound 13 TYR207—compound 13	1.95 5.00 4.20
14	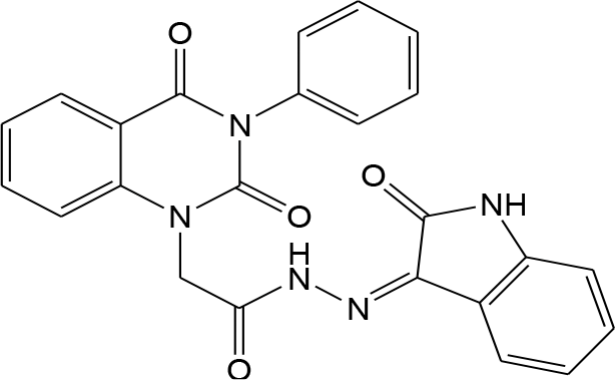	–8.7	**H-bonds** ARG253:NE—compound 14 THR318:OG1—compound 14 THR318:OG1—compound 14 **π**- **cation interactions** ARG253:NH1—compound 14 ARG253:NH1—compound 14	2.95 2.93 2.46 6.26 5.34
15	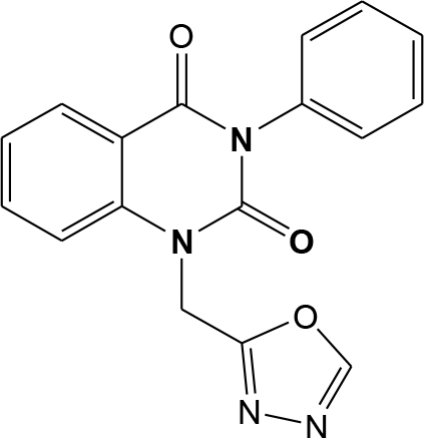	–9.1	**H-bonds** ASN274:ND2—compound 15 LYS429:NZ—compound 15 TYR528:OH—compound 15 TYR528:OH—compound 15 **π**- **π** **interactions** TYR528—compound 15 **π**- **cation interactions** LYS429:NZ—compound 15	2.95 2.64 2.80 2.85 5.52 4.60
16a	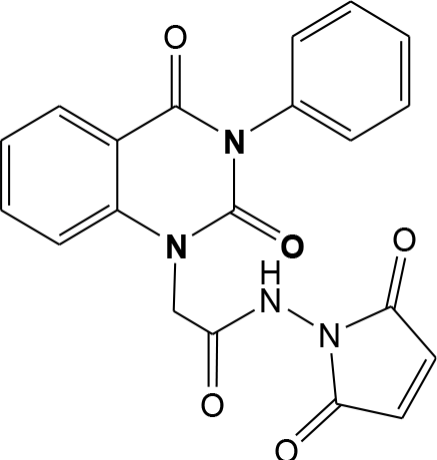	–8.1	**H-bonds** SER477:OG—compound 16a SER477:OG—compound 16a SER505:OG—compound 16a SER505:OG—compound 16a SER529:OG—compound 16a **π**- **π** **interactions** TYR528—compound 16a TYR528—compound 16a	2.87 2.96 2.93 2.69 3.10 5.00 4.03
16b	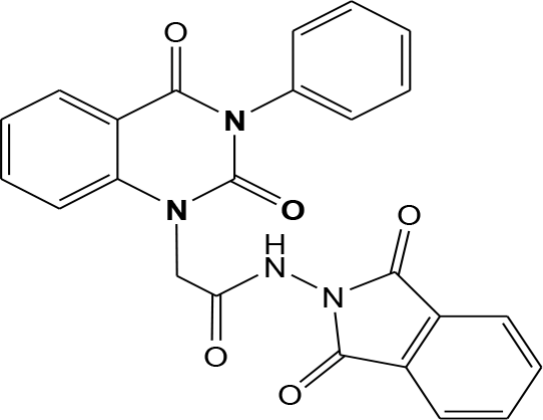	–8.8	**H-bonds** SER205:N—compound 16b SER205:OG—compound 16b ASN547:ND2—compound 16b ASN547:ND2—compound 16b **π**- **π** **interactions** TYR207—compound 16b **π**- **sigma interactions** TYR551:CD1—compound 16b	2.99 2.80 2.94 3.14 4.48 3.88
16c	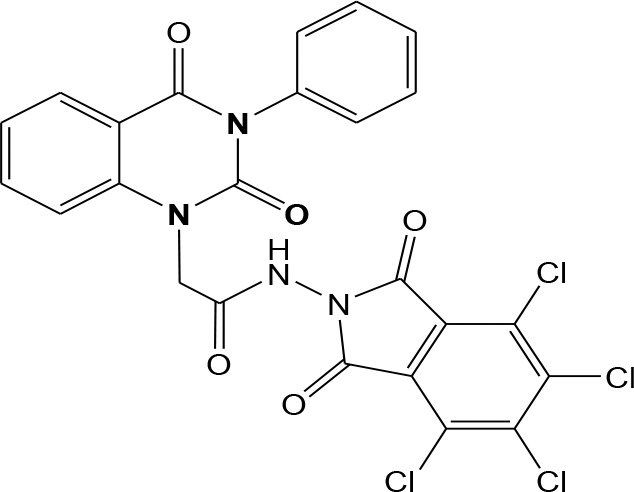	–8.6	**H-bonds** ASP204:N—compound 16c LYS305:NZ—compound 16c	2.95 3.14

*The structures of synthesized compounds **1-16** with the best binding energies are represented with their docking interactions, showing H-bonds, π-π, π-cation, and π-sigma interactions*.

**Table 2:** List of ADMET properties of synthesized hybrid molecules 1-16.

	**Molecular weight (g/mol)^a^**	**Blood-brain barrier (BBB+)^b^**	**Caco-2 permeability (Caco_2_+)^c^**	**%Human intestinal absorption (HIA+)^d^**	**AMES toxicity**	**Carcino- genicity**
**Reference range**	**130–725**	**−3–1.2**	**<25 is poor** **>500 is great**	**<25 is poor** **>80% is high**	**Non-toxic**	**Non-carcinogenic**
1	310.31	0.96	58.1	100.0	Non-toxic	Non-carcinogenic
2a	398.42	0.98	53.5	99.5	Non-toxic	Non-carcinogenic
2b	443.42	0.92	58.1	95.8	Non-toxic	Non-carcinogenic
2c	432.87	0.96	55.5	99.6	Non-toxic	Non-carcinogenic
2d	414.42	0.72	65.2	97.2	Non-toxic	Non-carcinogenic
2e	404.45	0.97	55.2	100.0	Non-toxic	Non-carcinogenic
3a	474.90	0.82	60.8	99.4	Non-toxic	Non-carcinogenic
4a	472.53	0.91	60.7	96.7	Non-toxic	Non-carcinogenic
5	376.37	0.96	54.1	100.0	Non-toxic	Non-carcinogenic
6	464.49	0.96	59.5	100.0	Non-toxic	Non-carcinogenic
7	380.38	0.97	57.7	98.5	Non-toxic	Non-carcinogenic
8	378.35	0.88	62.8	97.8	Non-toxic	Non-carcinogenic
9	386.80	0.87	61.7	100.0	Non-toxic	Non-carcinogenic
10	409.43	0.73	64.7	76.2	Non-toxic	Non-carcinogenic
11	392.38	0.96	63.1	100.0	Non-toxic	Non-carcinogenic
12	408.44	0.94	58.7	99.7	Non-toxic	Non-carcinogenic
13	409.43	0.73	64.7	76.2	Non-toxic	Non-carcinogenic
14	441.45	0.91	62.6	98.8	Non-toxic	Non-carcinogenic
15	320.31	0.99	50.6	100.0	Non-toxic	Non-carcinogenic
16a	390.36	0.997	56.4	100.0	Non-toxic	Non-carcinogenic
16b	440.42	0.97	56.8	99.6	Non-toxic	Non-carcinogenic
16c	578.20	0.94	60.7	97.7	Non-toxic	Non-carcinogenic

*The pharmacokinetic properties of compounds **1-16**, are predicted by admetSAR tool*.

a−d *total number of derivatives*.

**Table 3:** Physicochemical properties of the title compounds 1–16.

	**Logp**	**TPSA A^**2**^**	**HBA**	**HBD**	***N* rotatable**	**Volume A^**3**^**
**Reference range**	**<5**	**≤140**	**2.0–20.0**	**0.0–6.0**	**≤10**	
1	−0.30	99.13	7	3	3	267.62
2a	3.11	85.47	7	1	5	351.00
2b	3.07	131.30	10	1	6	374.34
2c	3.79	85.47	7	1	5	364.54
2d	3.05	105.70	8	2	5	359.02
2e	3.01	85.47	7	1	5	341.71
3a	2.51	93.42	6	1	5	395.86
4a	2.43	93.42	8	1	5	400.43
5	1.13	93.75	8	0	3	321.48
6	1.96	126.16	9	3	4	402.08
7	1.21	100.00	8	0	4	306.25
8	0.06	138.06	10	3	5	312.29
9	0.83	102.21	8	2	5	318.06
10	0.52	117.27	9	2	4	334.49
11	0.62	137.19	10	4	5	328.84
12	1.26	124.05	9	4	5	337.99
13	0.52	114.57	9	2	4	335.07
14	0.80	114.57	9	2	4	377.79
15	1.14	82.93	7	0	3	269.61
16a	0.69	112.18	9	1	4	323.66
16b	2.33	112.18	9	1	4	367.65
16c	4.18	112.18	9	1	4	421.79

*logp, logarithm of partition coefficient between n-octanol and water; TPSA, topological polar surface area; HBA, number of hydrogen bond acceptors; HBD, number of hydrogen bond donors; n rotatable, number of rotatable bonds*.

## Materials and Methods

The reagents used in this work were used without purification. The melting points of the prepared compounds were determined using Griffin apparatus and are uncorrected. The purity of the newly synthesized compounds **2-16** is mentioned by Thin Layer Chromatography TLC technique. IR spectra were recorded on Shimadzu 408 and Bruker Vect. 22. NMR spectra were run at 400 MHz using TMS as the internal standard. The chemical shifts were measured in ppm (δ) related to TMS (0.00 ppm). Finally, mass spectra were recorded on a HP model, Mass 5988 Mass spectrometer at 70 eV. All the prepared compounds were analyzed for C, H, and N at Cairo University, Egypt. Compounds **1** and **9** were already described (Hassan et al., 2013a).

### General Procedures for Synthesis of Schiff Bases 2a-e

2-(2,4-dioxo-3-phenyl-3,4-dihydroquinazolin-1(2H)-yl)acetohydrazide **1** (1 mmol) was heated under reflux for 8–12 h with the appropriate aromatic aldehydes namely benzaldehyde, 4-nitrobenzaldehyde, 4-chlorobenzaldehyde, salicylaldehyde, and thiophene-2-carboxaldehyde (1 mmol) in absolute ethanol (20 mL) and drops of piperidine as a catalyst. After cooling, the reaction mixture was filtered off and crystallized from appropriate solvents to afford the Schiff bases **2a-e**, respectively. ***2a*:** Gray crystals; yield 86%; mp 210–211°C. FT-IR (KBr, υ, cm^−1^) = 3,333 (NH), 1,625, 1,655 (C=O's), 1,576 (CH=N). ^1^H-NMR (DMSO d6, 400 MHz): δ (ppm) = 11.5 (s, 1H, NH), 7.2–8.06 (m, 14H, Ar-H), 5.8 (s, 1H, CH), 4.3 (s, 2H, CH_2_). ^13^C-NMR (CDCl_3_): δ 49.8, 114.4, 115.3, 124.7, 125.1, 126.5, 128.0, 128.2, 128.5, 128.7, 128.8, 128.9, 129.3, 129.5, 130.1, 131.8, 136.2, 136.6, 140.7, 142.5, 151.9, 161.3, 165.8. MS (EI): *m/z* (%) = 398.42 [M]^+^. *Anal*. Calcd for C_23_H_18_N_4_O_3_: C, 69.34; H, 4.55; N, 14.06%. Found C, 69.51; H, 4.72; N, 14.22%. ***2b*:** White crystals; yield 78%; mp 170–171°C. FT-IR (KBr, υ, cm^−1^) = 3,416 (NH), 1,640, 1,655 (C=O's). MS (EI): *m/z* (%) = 443.42 [M]^+^. *Anal*. Calcd for C_23_H_17_N_5_O_5_: C, 62.30; H, 3.86; N, 15.79%. Found C, 62.55; H, 4.05; N, 15.99%. ***2c*:** Yellow crystals; yield 69%; mp 230–231°C. FT-IR (KBr, υ, cm^−1^) = 3316 (NH), 1,640, 1,653 (C=O's). MS (EI): *m/z* (%) = 432.87 [M]^+^, 4.34.87 [M^+^+2] due to the presence of chlorine atom. *Anal*. Calcd for C_23_H_17_N_4_O_3_Cl: C, 63.82; H, 3.96; N, 12.94, Cl, 8.19%. Found C, 64.02; H, 4.07; N, 13.12%. ***2d*:** Faint yellow powder; yield 82%; mp 263–264°C. FT-IR (KBr, υ, cm^−1^) = 3,325 (NH), 1,640, 1,661 (C=O's). ^1^H-NMR (DMSO d6, 400 MHz): δ (ppm) =11.5 (s, 1H, NH), 7.2–8.06 (m, 13H, Ar-H), 6.4 (s, 1H, CH), 4.4 (s, 2H, CH_2_). MS (EI): *m/z* (%) = 414.42 [M]^+^. *Anal*. Calcd for C_23_H_18_N_4_O_4_: C, 66.66; H, 4.38; N, 13.52%. Found C, 66.81; H, 4.45; N, 13.72%. ***2e*:** White crystals; yield 81%; mp 190–191°C. FT-IR (KBr, υ, cm^−1^) = 3,168 (NH), 1,625, 1,649 (C=O's). MS (EI): *m/z* (%) = 404.45 [M]^+^. *Anal*. Calcd for C_21_H_16_N_4_O_3_S: C, 62.36; H, 3.99; N, 13.85; S, 7.93%. Found C, 62.52; H, 4.09; N, 14.12%.

### 2-(2,4-Dioxo-3-phenyl-3,4-dihydro-2H-quinazolin-1-yl)-N-(2-oxo-4-phenyl-azetidin-1-yl)-acetamide 3a

A mixture (2,4-Dioxo-3-phenyl-3,4-dihydro-2H-quinazolin-1-yl)-acetic acid benzylidene hydrazide **2a** (1 mmol) and Chloroacetyl chloride (1 mmol) in absolute ethanol (20 mL) was heated under refluxed for 10 h. After cooling, the reaction mixture was filtered off, dried then recrystallized from ethanol to give compound **3a** as gray crystals; yield 82%; mp 275–276°C. FT-IR (KBr, υ, cm^−1^) = 3,168 (NH), 1,555, 1,661 (C=O's). ^1^H-NMR (DMSO d6, 400 MHz): δ (ppm) = 11.5 (s, 1H, NH), 7.3–8.06 (m, 14H, Ar-H), 6.4 (d, 1H, CH-Ph), 5.5 (d, 1H, CH-Cl), 4.3 (s, 2H, CH_2_). ^13^C-NMR (CDCl_3_): δ 49.8, 58.1, 59.5, 114.3, 115.2, 124.7, 125.0, 126.5, 126.7, 126.9, 127.1, 128.5, 128.6, 128.8, 128.9, 129.1, 129.3, 131.8, 136.2, 139.5, 140.7, 151.9, 161.2, 165.8, 167.2. MS (EI): *m/z* (%) = 474.91 [M]^+^, 476.91 [M^+^+2] due to the presence of chlorine atom. *Anal*. Calcd for C_25_H_19_N_4_O_4_Cl: C, 63.23; H, 4.03; N, 11.80%. Found C, 63.54; H, 4.18; N, 12.08%.

### 2-(4-Methylene-2-oxo-3-phenyl-3,4-dihydro-2H-quinazolin-1-yl)-N-(4-oxo-4-phenyl-thiazolidin-3-yl)-acetamide 4a

A mixture of Schiff base **2a** (1 mmol) and thioglycolic acid (1 mmol) in ethanol was heated under refluxed for 7 h. After cooling, the solid formed was filtered and purified by recrystallization from ethanol to give pure compound **4a** as yellow brownish crystals; yield 76%; mp 255–256°C. FT-IR (KBr, υ, cm^−1^) = 3,477 (NH), 1,653, 1,725 (C=O's). ^1^H-NMR (DMSO d6, 400 MHz): δ (ppm) = 11.5 (s, 1H, NH), 7.2–8.06 (m, 14H, Ar-H), 6.4 (s, 1H, CH-Ph), 5.4 (s, 2H, CH_2_), 4.3 (s, 2H, CH_2_). ^13^C-NMR (CDCl_3_): δ 33.9, 49.8, 66.2, 114.4, 115.3, 124.7, 125.1, 126.5, 128.0, 128.1, 128.2, 128.4, 128.6, 128.8, 128.9, 129.3, 129.5, 131.8, 136.2, 137.0, 140.7, 151.9, 161.3, 165.8, 167.3. MS (EI): *m/z* (%) = 472.53 [M]^+^. *Anal*. Calcd for C_25_H_20_N_4_O_4_S: C, 63.55; H, 4.27; N, 11.86%. Found C, 63.77; H, 4.35; N, 12.12%.

### 4-Methylene-1-[2-(3-methyl-5-oxo-4,5-dihydro-pyrazol-1-yl)-2-oxo-ethyl]-3-phenyl-3,4-dihydro-1H-quinazolin-2-one 5

A solution of hydrazide hydrate **1** (1 mmol) and ethyl acetoacetate (1.1 mmol) in ethanol was refluxed for 8 h. After completion the reaction (monitored by TLC), the mixture was filtered off and then recrystallized from benzene to afford the product **5** as yellow crystals; yield 84%; mp 215–216°C. FT-IR (KBr, υ, cm^−1^) = 3,245 (NH), 1,605, 1,649 (C=O's). ^1^H-NMR (DMSO d6, 400 MHz): δ (ppm) = 7.2–8.07 (m, 9H, Ar-H), 4.4 (s, 4H, 2CH_2_), 1.4 (s, 3H, CH_3_). MS (EI): *m/z* (%) = 376.80 [M]^+^. *Anal*. Calcd for C_20_H_16_N_4_O_4_: C, 63.83; H, 4.28; N, 14.89%. Found C, 64.03; H, 4.35; N, 15.17%.

### 3-Amino-1-[2-(2,4-dioxo-3-phenyl-3,4-dihydro-2H-quinazolin-1-yl)-acetyl]-5-phenyl-2,3-dihydro-1H-pyrazole-4-carbonitrile 6

A mixture of hydrazide **1** (1 mmol) and benzylidene malononitrile (1 mmol) in ethanol was heated under reflux for 8 h. After cooling, the solid formed was filtered off and dried then recrystallized from benzene to yield compound **6** as white crystals; yield 83%; mp 287–288°C. FT-IR (KBr, υ, cm^−1^) = 3,416 (NH), 2,225 (CN), 1,649, 1,731 (C=O's). ^1^H-NMR (DMSO d6, 400 MHz): δ (ppm) =11.5 (s, 1H, NH), 7.2–8.06 (m, 14H, Ar-H), 8.7 (s, 2H, NH_2_), 4.3 (s, 2H, CH_2_). ^13^C-NMR (CDCl_3_): δ 49.8, 79.2, 114.4, 115.3, 115.9, 124.7, 125.1, 126.5, 126.7, 126.8, 128.4, 128.5, 128.6, 128.8, 128.9, 129.2, 129.3, 129.5, 131.8, 131.9, 136.2, 137.5, 140.7, 151.9, 161.3, 167.3. MS (EI): *m/z* (%) = 464.49 [M]^+^. *Anal*. Calcd for C_26_H_20_N_6_O_3_: C, 67.23; H, 4.34; N, 18.09%. Found C, 67.53; H, 4.55; N, 18.22%.

### 1-[2-(5-Mercapto-[1,3,4]oxadiazol-2-yl)-2-oxo-ethyl]-3-phenyl-dihydro-pyrimidine-2,4-dione 7

To an ethanolic solution of hydrazide **1** (1 mmol), a 20 mL of carbon disulfide was added then refluxed for 5 h. After completion the reaction (monitored by TLC), the reaction mixture was filtered off, dried then recrystallized from ethanol to afford pure product **7** as yellow crystals; yield 81%; mp 215–217°C. FT-IR (KBr, υ, cm^−1^) = 3,416 (NH), 1,623, 1,663 (C=O's). ^1^H-NMR (DMSO d6, 400 MHz): δ (ppm) = 7.2–8.08 (m, 9H, Ar-H), 4.7 (s, 1H, SH), 4.3 (s, 2H, CH_2_). MS (EI): *m/z* (%) = 380.38 [M]^+^. *Anal*. Calcd for C_18_H_12_N_4_O_4_S: C, 56.84; H, 3.18; N, 14.73%. Found C, 57.13; H, 3.25; N, 14.92%.

### 1-[2-(5-Hydrazino-[1,3,4]oxadiazol-2-yl)-2-oxo-ethyl]-3-phenyl-1H-quinazoline-2,4-dione 8

Two gram of later product **7** was allowed to react with 4 ml hydrazine in ethanol under reflux for 7 h. After cooling, the solid formed was filtered off, dried then recrystallized from ethanol to give compound 8 as white crystals; yield 79%; mp 210–211°C. FT-IR (KBr, υ, cm^−1^) = 3,420 (NH), 1,623, 1,661 (C=O's). ^1^H-NMR (DMSO d6, 400 MHz): δ (ppm) =11.5 (s, 1H, NH), 8.7 (s, 2H, NH_2_), 7.2–8.06 (m, 9H, Ar-H), 4.3 (s, 2H, CH_2_). ^13^C-NMR (CDCl_3_): δ 54.4, 114.4, 115.3, 124.7, 125.1, 126.5, 128.6, 128.8, 129.3, 129.5, 131.8, 136.2, 140.7, 151.9, 161.3, 162.3, 161.8, 194.5. MS (EI): *m/z* (%) = 378.35 [M]^+^. *Anal*. Calcd for C_18_H_14_N_6_O_4_: C, 57.14; H, 3.73; N, 22.21%. Found C, 57.33; H, 4.05; N, 22.52%.

### N-(2-Imino-4-oxo-thiazolidin-3-yl)-2-(2-oxo-3-phenyl-3,4-dihydro-2H-quinazolin-1-yl)-acetamide 10

To an ethanolic solution of chloro-acetic acid N'-[2-(2,4-dioxo-3-phenyl-3,4-dihydro-2H-quinazolin-1-yl)-acetyl]-hydrazide **9** (1 mmol), potassium thiocyanate (1 mmol) was added then the reaction mixture was refluxed for 5 h. After cooling, the solid precipitated was filtered off and recrystallized from ethanol to yield compound **10** as white crystals; yield 80%; mp 255–257°C. FT-IR (KBr, υ, cm^−1^) = 3,483 (NH), 1,621, 1,675 (C=O's). ^1^H-NMR (DMSO d6, 400 MHz): δ (ppm) =11.5 (s, 2H, 2NH), 7.2–7.9 (m, 9H, Ar-H), 5.6 (s, 2H, CH_2_), 4.3 (s, 2H, CH_2_). ^13^C-NMR (CDCl_3_): δ 33.9, 49.8, 114.4, 115.3, 124.7, 125.1, 126.5, 128.6, 128.8, 129.3, 129.5, 131.8, 136.2, 140.7, 151.9, 160.3, 161.3, 165.8, 167.3. MS (EI): *m/z* (%) = 409.43 [M]^+^. *Anal*. Calcd for C_19_H_15_N_5_O_4_S: C, 55.74; H, 3.69; N, 17.11%. Found C, 56.03; H, 3.85; N, 17.32%.

### (4-Methylene-2-oxo-3-phenyl-3,4-dihydro-2H-quinazolin-1-yl)-acetic acid N′-(2-amino-oxazol-4-yl)-hydrazide 11

A mixture of compound **9** (1 mmol) in dioxane and urea (1 mmol) was refluxed for 10 hr. Afterward, the formed precipitate was filtered and purified by recrystallization from benzene to give pure product **11** as yellow crystals; yield 86%; mp 245–246°C. FT-IR (KBr, υ, cm^−1^) = 3,378 (NH), 1,617, 1,645 (C=O's). ^1^H-NMR (DMSO d6, 400 MHz): δ (ppm) =11.5 (s, 1H, NH), 10.5 (s, 1H, NH), 8.7 (s, 2H, NH_2_), 7.2–8.06 (m, 9H, Ar-H), 4.3 (s, 4H, 2CH_2_). MS (EI): *m/z* (%) = 392.38 [M]^+^. *Anal*. Calcd for C_19_H_16_N_6_O_4_: C, 58.16; H, 4.11; N, 21.42%. Found C, 58.33; H, 4.32; N, 21.64%.

### (4-Methylene-2-oxo-3-phenyl-3,4-dihydro-2H-quinazolin-1-yl)-acetic acid N′-(2-amino-thiazol-4-yl)-hydrazide 12

A mixture of compound **9** (1 mmol) in dioxane and thiourea (1 mmol) was heated under reflux for 10 h. Afterward, the formed precipitate was filtered and purified by recrystallization from benzene to give pure compound **12** as white crystals; yield 86%; mp 190–192°C. FT-IR (KBr, υ, cm^−1^) = 3,378 (NH), 1,617, 1,645 (C=O's). ^1^H-NMR (DMSO d6, 400 MHz): δ (ppm) =11.5 (s, 1H, NH), 10.5 (s, 1H, NH), 8.7 (s, 2H, NH_2_), 7.2–8.06 (m, 9H, Ar-H), 4.3 (s, 4H, 2CH_2_). MS (EI): *m/z* (%) = 408.44 [M]^+^. *Anal*. Calcd for C_19_H_16_N_6_O_3_S: C, 55.87; H, 3.95; N, 20.58%. Found C, 56.15; H, 4.08; N, 20.72%.

### 4-Methylene-2-oxo-3-phenyl-3,4-dihydro-2H-quinazolin-1-yl)-acetic acid (4-oxo-thiazolidin-2-ylidene)-hydrazide 13

A solution of compound **9** (1 mmol) and ammonium thiocyanate (1 mmol) in ethanol was refluxed for 12 h. After completion the reaction (monitored by TLC), the reaction mixture was filtered off, dried then recrystallized from ethanol to afford pure product **13** as white crystals; yield 82%; mp 290–291°C. FT-IR (KBr, υ, cm^−1^) = 3,316 (NH), 1,625, 1,674 (C=O's). ^1^H-NMR (DMSO d6, 400 MHz): δ (ppm) =11.3 (s, 2H, 2NH), 7.2–8.07 (m, 9H, Ar-H), 4.3 (s, 4H, 2CH_2_). MS (EI): *m/z* (%) = 409.43 [M]^+^. *Anal*. Calcd for C_19_H_15_N_5_O_4_S: C, 55.74; H, 3.69; N, 17.77%. Found C, 55.93; H, 3.91; N, 17.96%.

### [3-(1-Methyl-buta-1,3-dienyl)-2,4-dioxo-3,4-dihydro-2H-quinazolin-1-yl]-acetic acid (2-oxo-1,2,3a,7a-tetrahydro-indol-3-ylidene)-hydrazide 14

To an ethanolic solution of compound **9** (1 mmol), Isatine (1 mmol) was added and the reaction mixture was heated under reflux for 8 h. After cooling; a solid product was filtered off and recrystallized from ethanol/benzene to give compound **14** as yellow crystals; yield 86%; mp 300–301°C. FT-IR (KBr, υ, cm^−1^) = 3,236 (NH), 1,643, 1,655 (C=O's). ^1^H-NMR (DMSO d6, 400 MHz): δ (ppm) =11.5 (s, 1H, NH), 8.7 (s, 1H, NH), 7.2–8.0 (m, 13H, Ar-H), 4.3 (s, 2H, CH_2_). ^13^C-NMR (CDCl_3_): δ 49.8, 110.6, 114.4, 115.3, 121.9, 123.1, 124.7, 125.1, 126.5, 128.6, 128.8, 129.3, 129.5, 129.6, 131.8, 132.0, 136.2, 140.7, 147.2, 151.9, 153.8, 161.3, 165.8, 167.3. MS (EI): *m/z* (%) = 441.45 [M]^+^. *Anal*. Calcd for C_24_H_19_N_5_O_4_: C, 65.30; H, 4.34; N, 15.86%. Found C, 65.51; H, 4.45; N, 16.02%.

### 4-Methylene-1-[1,3,4] oxadiazol-2-ylmethyl-3-phenyl-3,4-dihydro-1H-quinazolin-2-one 15

A mixture of hydrazide **1** (1 mmol) and triethyl orthoformate (1.1 mmol) in ethanol was heated reflux for 8 h. After cooling; the formed precipitate was filtered and purified by crystallization from benzene to give product 15 as yellow crystals; yield 80%; mp 200–201°C. FT-IR (KBr, υ, cm^−1^) = 3,236 (NH), 1,628, 1,685 (C=O's). ^1^H-NMR (DMSO d6, 400 MHz): δ (ppm) = 7.2–8.06 (m, 10H, Ar-H+CH), 4.3 (s, 2H, CH_2_). MS (EI): *m/z* (%) = 320.31 [M]^+^. *Anal*. Calcd for C_17_H_12_N_4_O_3_: C, 63.75; H, 3.78; N, 17.49%. Found C, 63.93; H, 4.01; N, 17.62%.

### General Procedure for Synthesis of Imide 16a-c

(4-Methylene-2-oxo-3-phenyl-3,4-dihydro-2*H*-quinazolin-1-yl)-acetic acid hydrazide **1** (1 mmol) was heated under reflux for 8–12 h with the appropriate anhydrides such as maleic, phthalic, and tetrachlorophthalic anhydrides, respectively (1 mmol) in glacial acetic acid (20 mL). After cooling, the reaction mixture was filtered off and crystallized from appropriate solvents to afford the imide **16**a-c, respectively. ***16a*:** yield 80%; mp 268–270°C. FT-IR (KBr, υ, cm^−1^) = 3,326 (NH), 1,610, 1,678 (C=O's). ^13^C-NMR (CDCl_3_): δ 49.8, 114.4, 115.3, 124.7, 125.1, 126.5, 128.6, 128.8, 129.3, 129.5, 131.8, 133.2, 133.4, 136.2, 140.7, 151.9, 161.3, 165.8, 168.2, 168.4. MS (EI): *m/z* (%) = 390.36 [M]^+^. *Anal*. Calcd for C_20_H_14_N_4_O5: C, 61.54; H, 3.62; N, 14.35%. Found C, 61.73; H, 3.85; N, 14.52%. ***16b*:** yield 86%; mp 262–264°C. FT-IR (KBr, υ, cm^−1^) = 3,351 (NH), 1,645, 1,667 (C=O's). MS (EI): *m/z* (%) = 440.42 [M]^+^. *Anal*. Calcd for C_24_H_16_N_4_O_5_: C, 65.45; H, 3.66; N, 12.72%. Found C, 65.63; H, 3.81; N, 12.94%. ***16c*:** yield 86%; mp 280–282°C. FT-IR (KBr, υ, cm^−1^) = 3,316 (NH), 1,663, 1,685 (C=O's). ^1^H-NMR (DMSO d6, 400 MHz): δ (ppm) =11.5 (s, 1H, NH), 7.2–8.06 (m, 9H, Ar-H), 4.3 (s, 2H, CH_2_). MS (EI): *m/z* (%) = 576.20 [M]^+^, 578.20 [M^+^+2], 580.20 [M^+^+4], 582.20 [M^+^+6], due to the presence of four chlorine atoms. *Anal*. Calcd for C_24_H_12_N_4_O_5_Cl_4_: C, 49.86; H, 2.09; N, 9.69%. Found C, 50.09; H, 2.15; N, 9.82%.

### *In silico* Docking

The chemical structures of the synthesized 22 molecules are drawn in cdx format using Chem Draw Ultra v.7.0.1, and the obtained were then converted to SDF format. In-house database of 22-small molecules is constructed. All the atomic coordinates were changed to pdbqt set-up using Open Babel GUI. The X-ray crystal structure of enzyme for this study was availed from RCSB Protein Data Bank (www.rscb.org/pdb/). The protonation states of the residues were theoretically calculated using PDB2PQR (Dolinsky et al., 2007) and Propka 3.1 (Olsson et al., 2011) servers. Furthermore, the three-dimensional 3D coordinates for the target enzyme and ligand molecules were energy minimized using CHARMm Force Field (Brooks et al., 2009) in Discovery Studio and AMBER Force Field (Salomon-Ferrer et al., 2013) in Open Babel, respectively (with a limit of 500 steps of steepest algorithm for each ligand), to get more stable confirmations then used for *in silico* screening approach. The grid size of dimensions 25 × 25 × 25 Å^3^ is set to cover all binding site region. Ligand-enzyme docking simulations are conducted using PyRx tool, Autodock 4.2 to find the best-fit orientation of a compound that bind to the target enzyme. The docking studies using Autodock 4.2 of PyRx 8.0 tool uses the binding free energy calculations to identify the best binding pattern of the ligand molecule to the active site region of the target. Understanding of the intermolecular interactions between ligand molecules and enzyme is a key for development of novel therapeutic agents. Three-dimensional 3D pose views of the ligands-enzyme complexes are visualized using Accelrys discovery studio 3.5 (Accelrys Discovery Studio Visualizer software 2010). The visualization of electrostatic potential of the molecules was obtained using Gaussian 03 program package, through PM6 semi empirical method (Stewart, 1989). The molecular parameters of the synthesized compounds were checked by Mol inspiration server https://molinspiration.com/ to fit into Lipinski rule of Five (Ro5), which is a key way to satisfy the rational drug design and to calculate the bioactivity score for drug meant for oral use. The drug-likeness was evaluated by helping of the following attributes: hydrogen bond donor HBD (not more than 5), hydrogen bond acceptor HBA (not more than 10), the topological surface areas TPSA (not more than 140 Å^2^), partition coefficient LogP (not more than 5), and rotatable bonds (not more than 10). Moreover, to further estimate the drug ability of the target compounds, we herein reported their ADMET properties by using admetSAR server http://lmmd.ecust.edu.cn/admetsar1/.

## Conclusion

In summary, the efficient synthesis of novel series of quinazolin-2,4-dione analogs coupled to N-heterocyclic moieties *via* acetyl/amide linkage is described. *In silico* screening was performed for searching about potent *pf*DHODH inhibitors. Docking of the target analogs to *pf*DHODH yielded binding energy between −12.2 and −5.7 kcal/mol, compared to −7.2 kcal/mol for reference drug (Chloroquine), and disclosed the analog **11** as a possible lead compound. The obtained results revealed that the newly synthesized compounds represent promising starting points for development of novel drug candidates against malaria.

## Data Availability Statement

All datasets generated for this study are included in the article/Supplementary Material.

## Author Contributions

All authors designed the study, contributed to the revision of the drafts, and agreed on the final version to be submitted. AA, MM, and AM were responsible for synthesis of compounds. AA did the virtual screening approach and he wrote the preliminary draft of the manuscript. AM, ME-N, and HT were responsible for the spectral analysis section.

## Conflict of Interest

The authors declare that the research was conducted in the absence of any commercial or financial relationships that could be construed as a potential conflict of interest.

## Abbreviations

^1^H-NMR: Proton- Nuclear Magnetic Resonance
*pf*DHODH: *Plasmodium falciparum* Dihydroorotate dehydrogenase
ESP: Electrostatic potential
3D: Three-dimensional
ADME: Adsorption, distribution, metabolic and excretion.
