# Supplementary material for: Novel Quinazolin-2,4-Dione Hybrid Molecules as Possible Inhibitors Against Malaria: Synthesis and in silico Molecular Docking Studies
Source: Front Mol Biosci. 2020 Jun 5;7:105. doi: 10.3389/fmolb.2020.00105 (PMC7291371; doi:10.3389/fmolb.2020.00105)
Supplement: Supplementary file 1 [file Data_Sheet_1.PDF]

Novel quinazolin-2,4-dione hybrid molecules as possible inhibitors against Malaria: Synthesis and In silico molecular docking studies

Aboubakr Haredi Abdelmonsef [1]*, Mahmoud Eldeeb Mohamed [1], Mohamed El-Naggar [2], Hussain Temairk [1], Ahmed Mohamed Mosallam[1]

[1]Chemistry Department, Faculty of Science, South Valley University, 83523 Qena, Egypt

[2]Chemistry Department, Faculty of Sciences, University of Sharjah, 27272 Sharjah, UAE

*Email: aboubakr.ahmed@sci.svu.edu.eg

Figure S1. Three- dimensional structure of the target enzyme *pf*DHODH

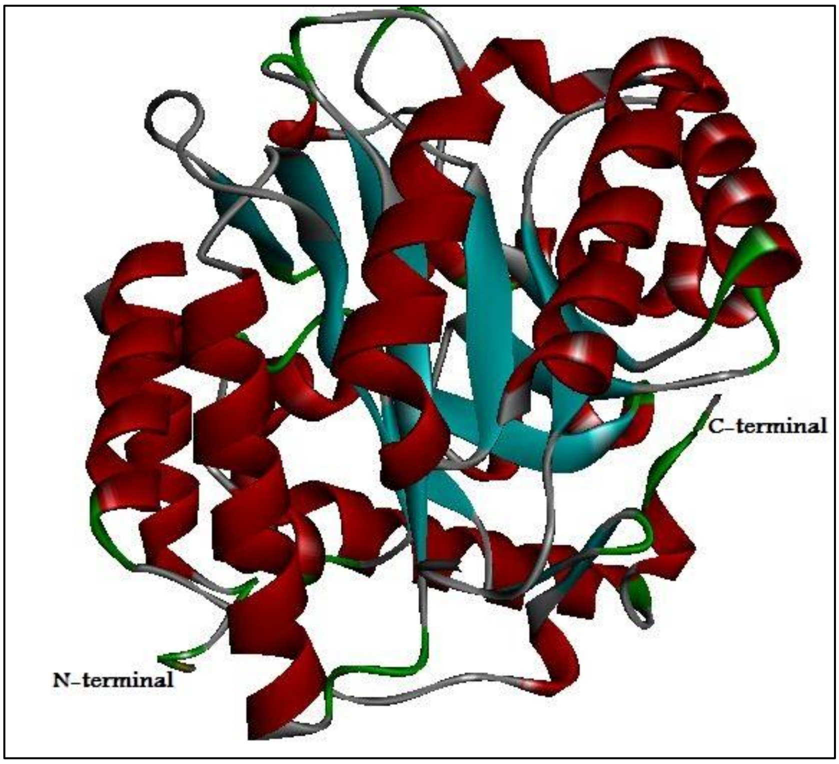

The X-ray crystal structure of enzyme obtained from RCSB Protein Data Bank

Figure S2. Three- dimensional structures of the docked molecules

Compound 1

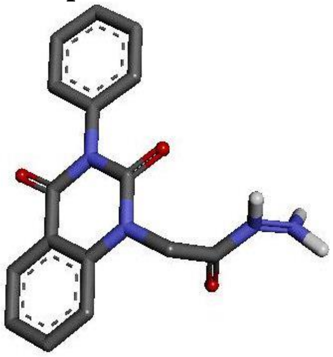

Compound 2a

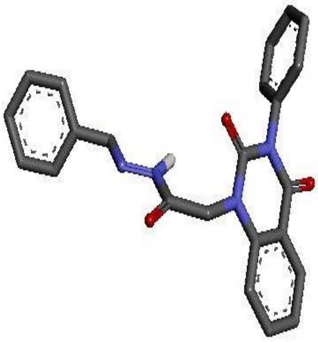

Compound 2b

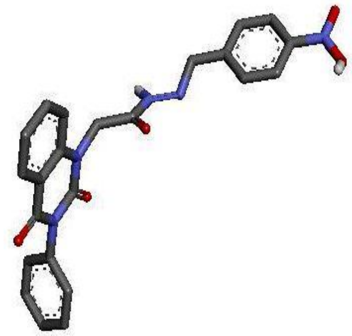

Compound 2c

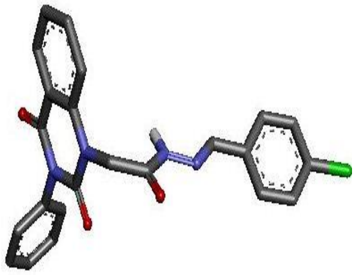

Compound 2d

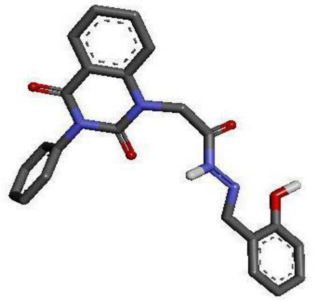

Compound 2e

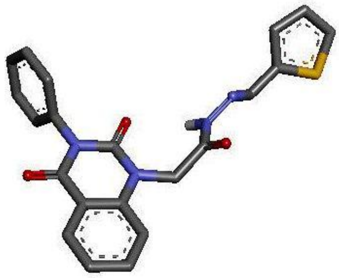

Compound 3a

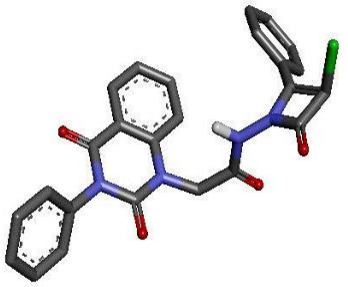

Compound 4a

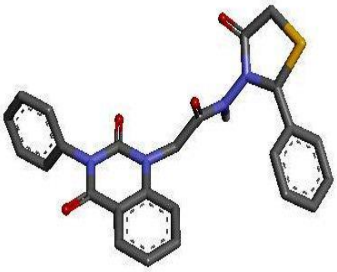

Compound 5

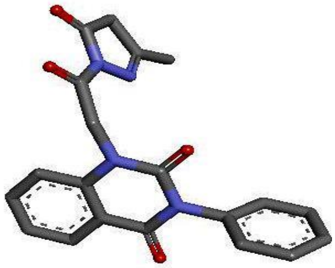

Compound 6

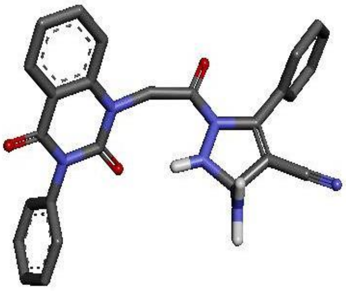

Compound 7

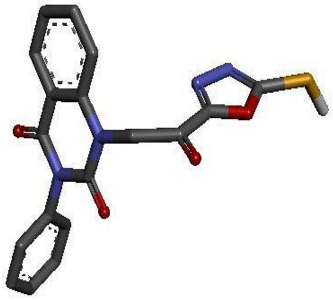

Compound 8

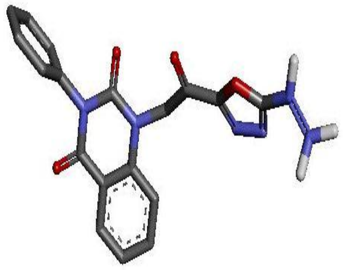

Compound 9

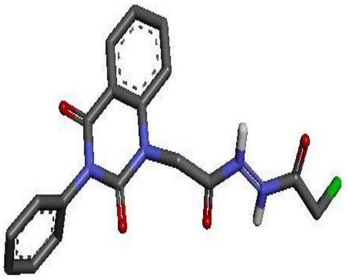

Compound 10

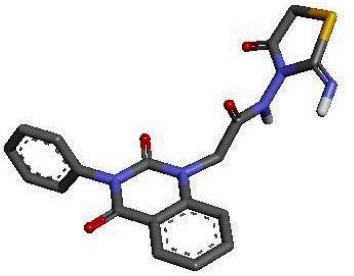

Compound 11

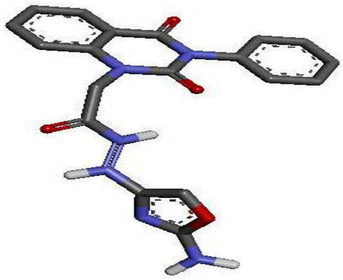

Compound 12

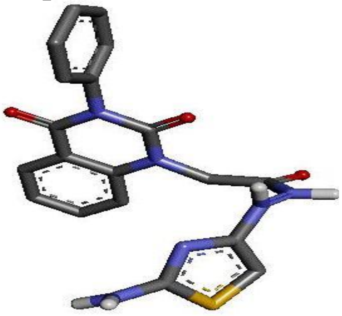

Compound 13

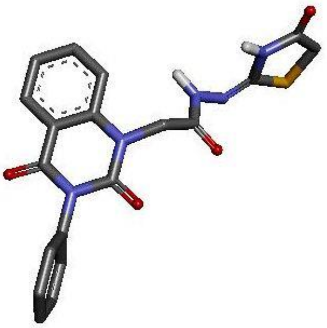

Compound 14

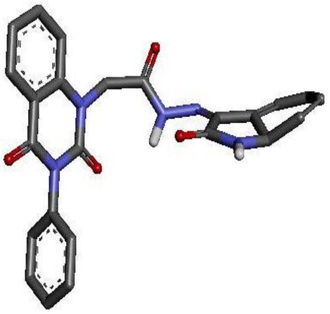

Compound 15

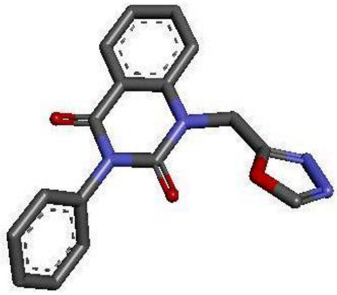

Compound 16a

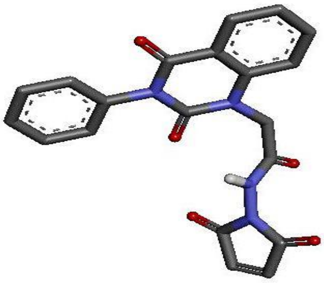

Compound 16b

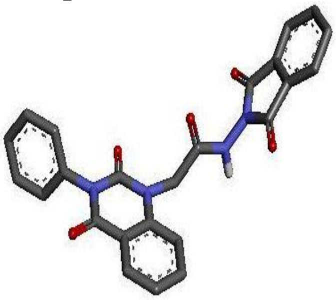

Compound 16c

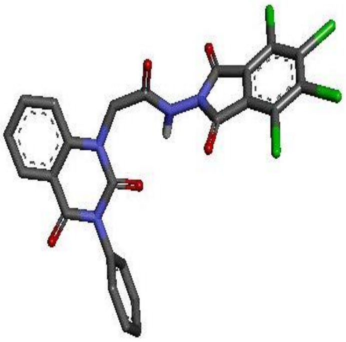

Figure S3. Molecular interactions between the rest compounds and *pf*DHODH

Compound 2a

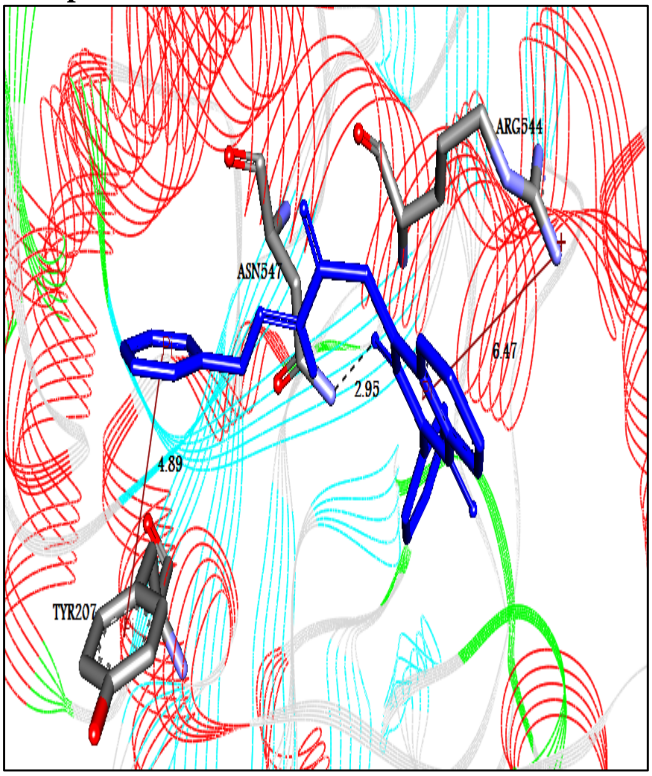

Compound 2b

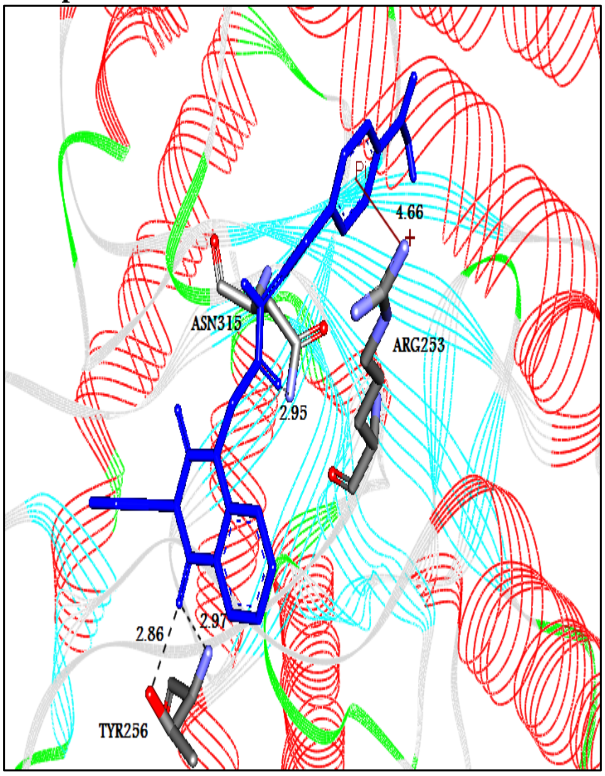

Compound 2c

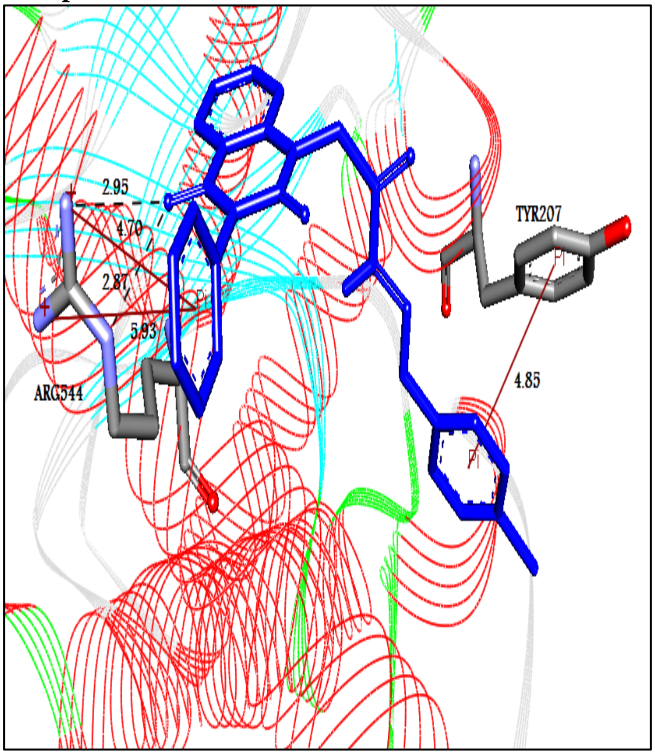

Compound 2e

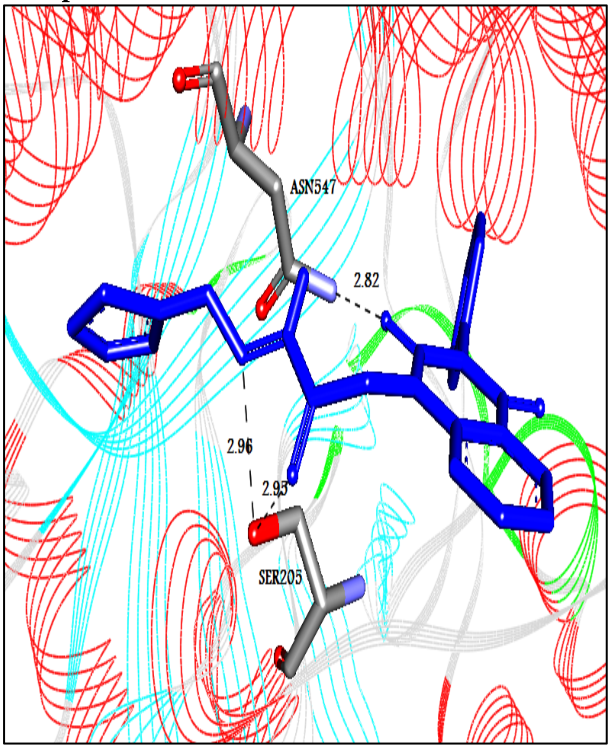

Compound 3a

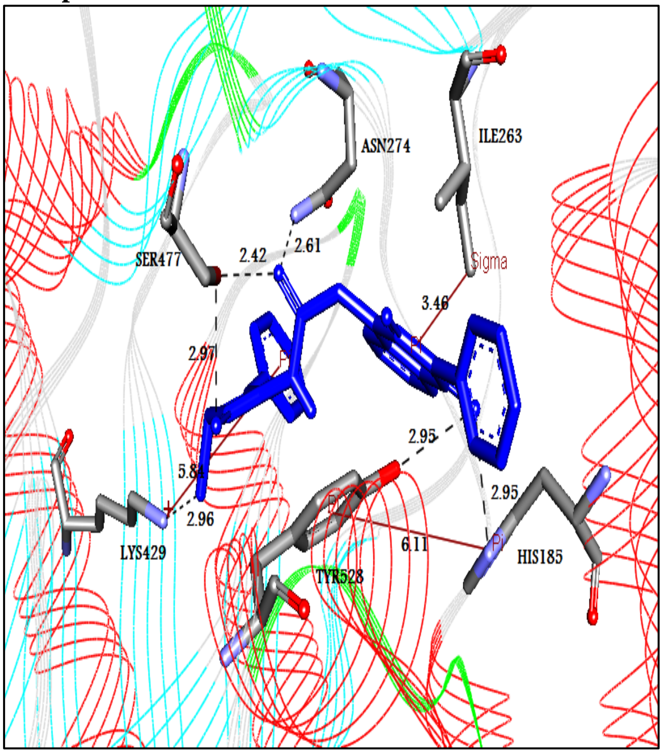

Compound 4a

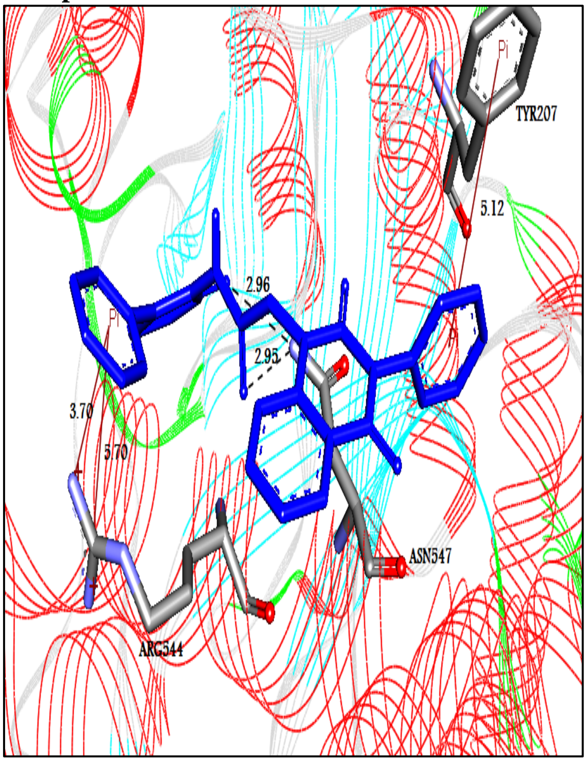

Compound 5

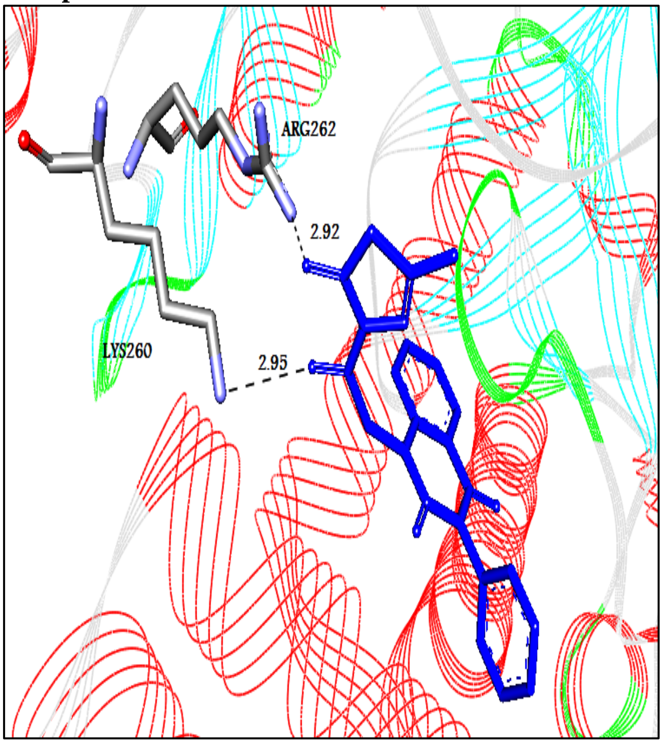

Compound 6

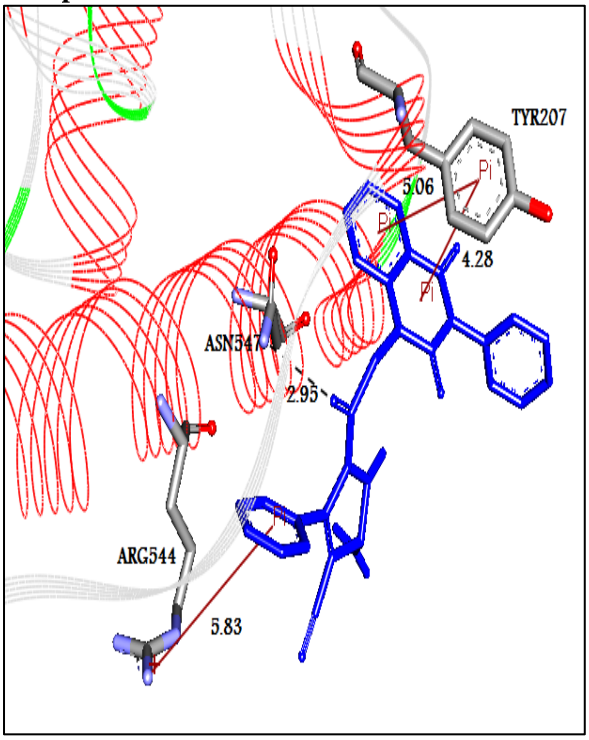

Compound 7

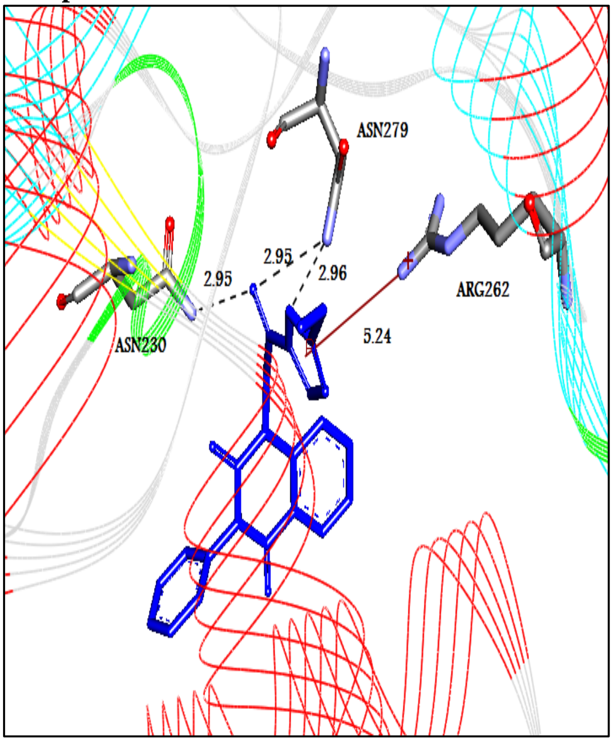

Compound 8

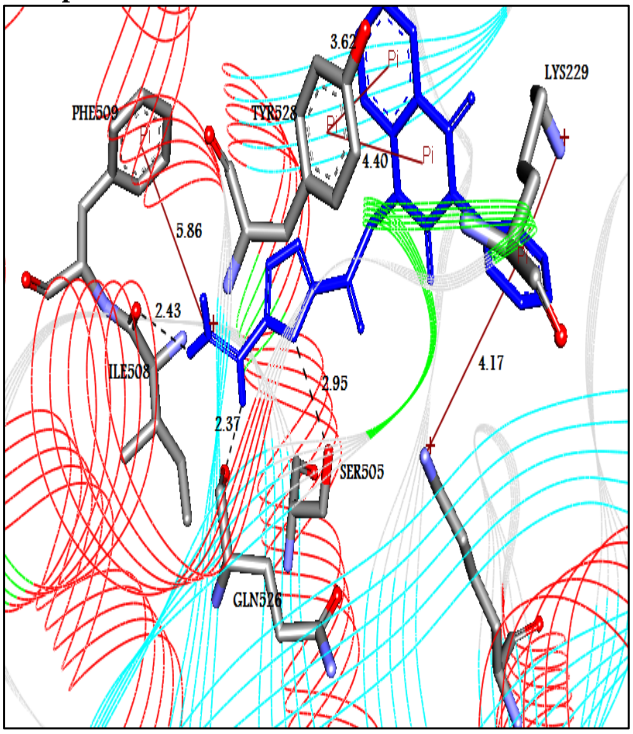

Compound 9

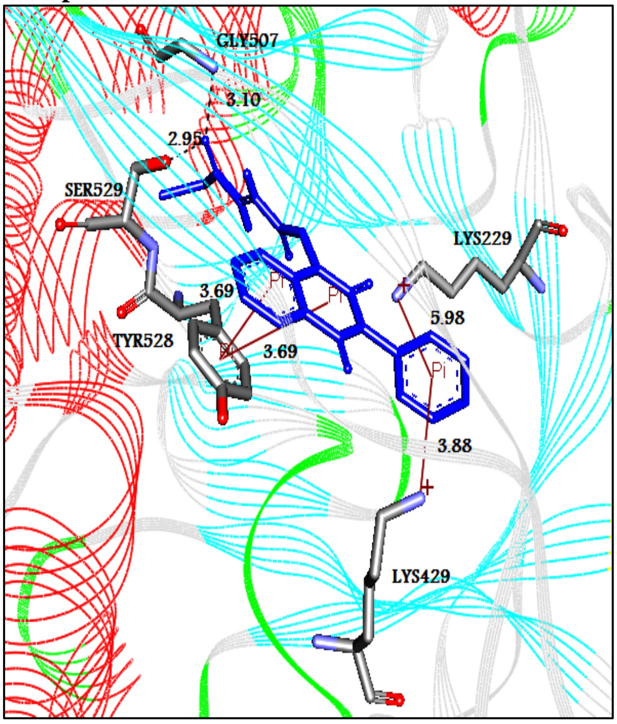

Compound 10

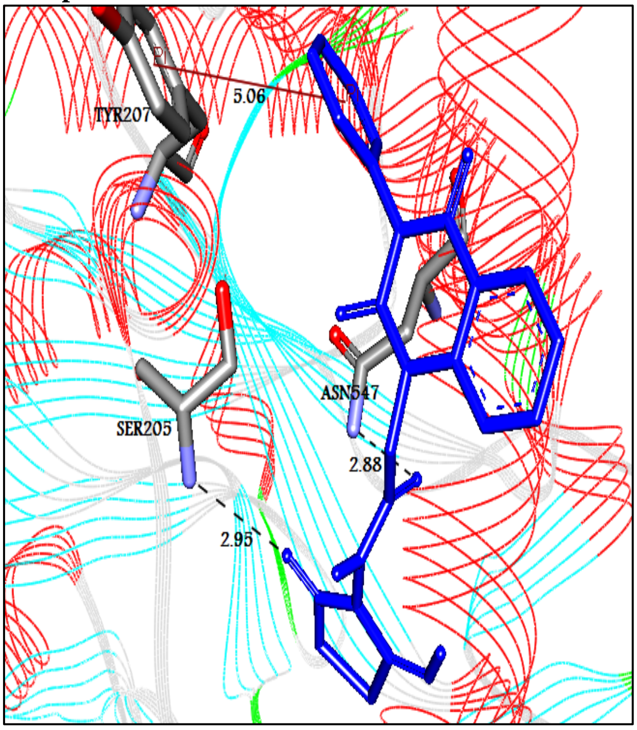

Compound 12

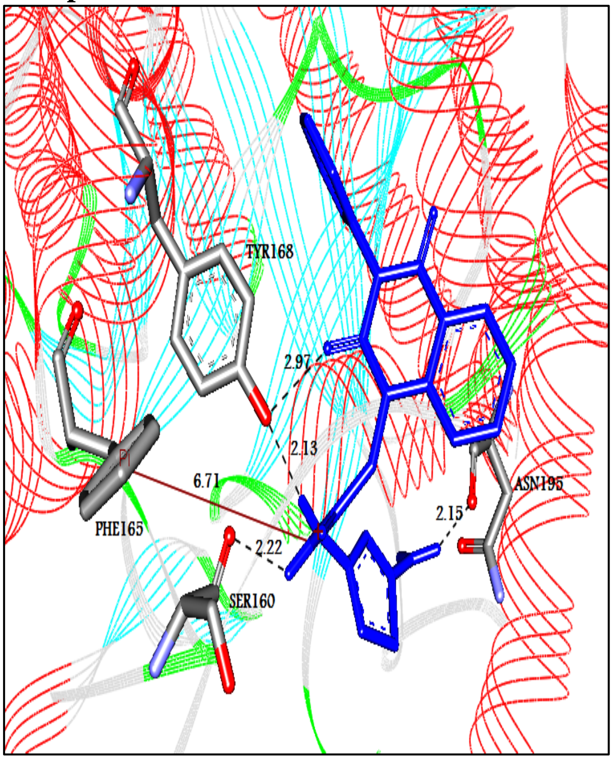

Compound 13

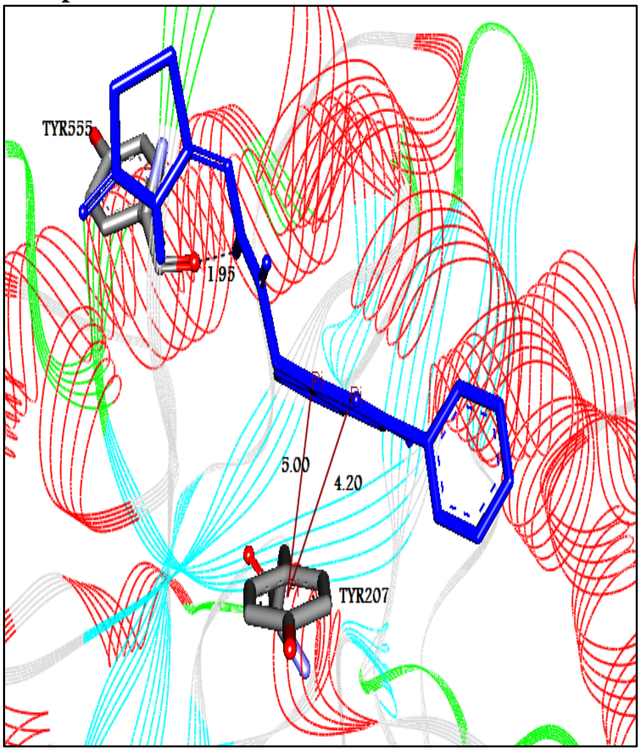

Compound 14

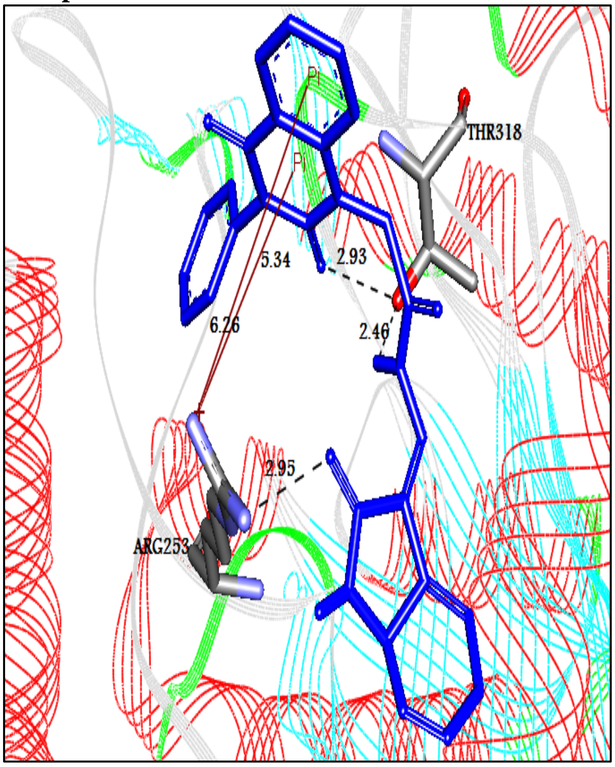

Compound 15

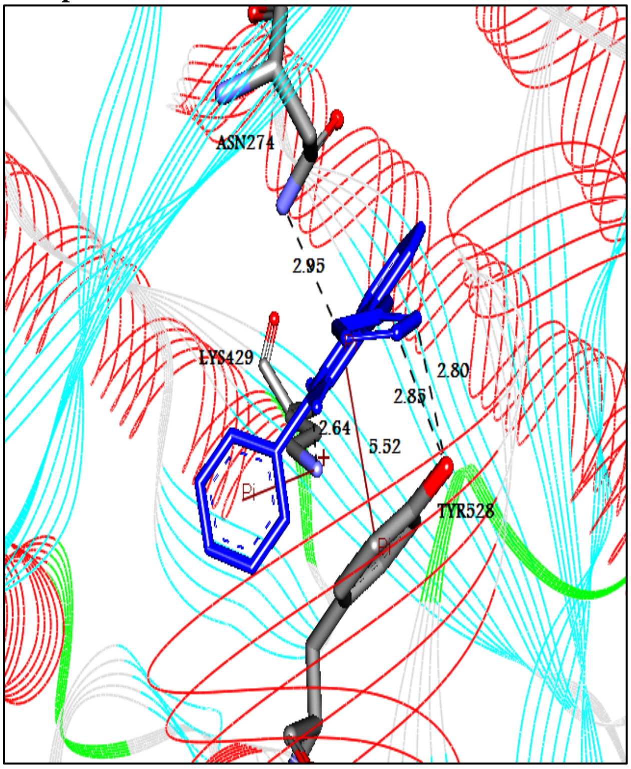

Compound 16a

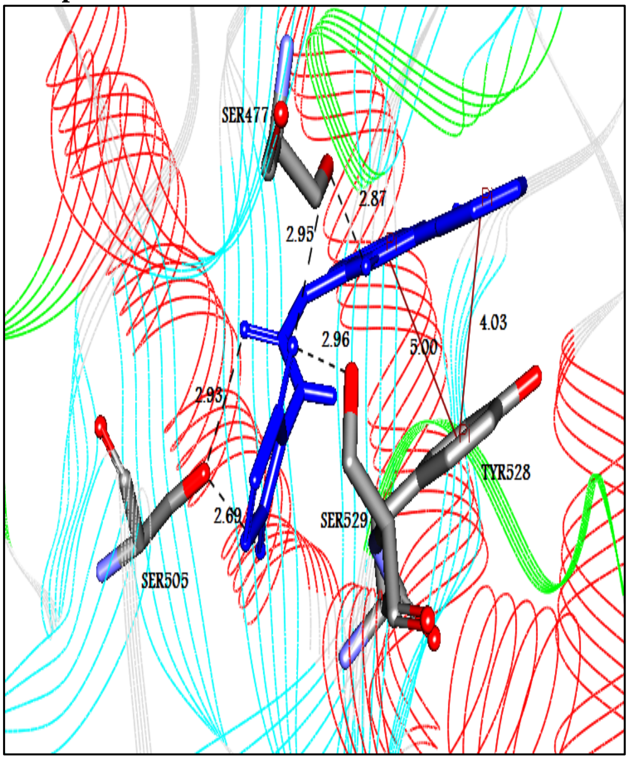

Compound 16b

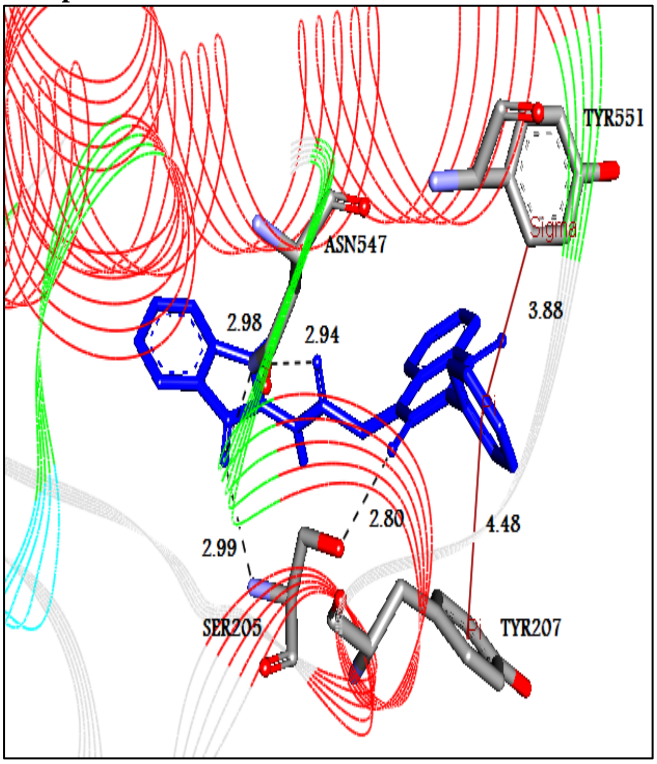

Compound 16c

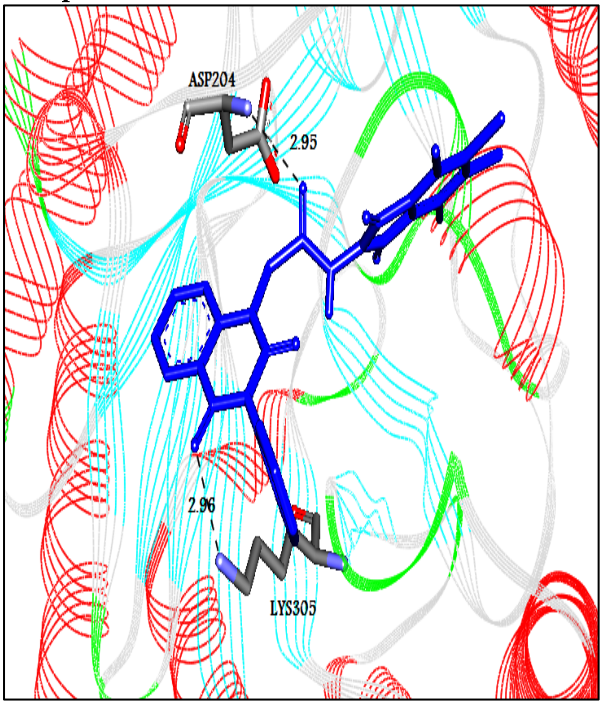

Three-dimensional of docking pose of the rest compounds in the active pocket of *pf*DHODH. Blue stick models represented the docked compounds, and gray models represented the active site region. H-bond interactions are shown in black dotted lines. π- interactions are shown in brown lines.

Figure S4. **The electrostatic potential maps of the rest compounds.**

Compound 2a

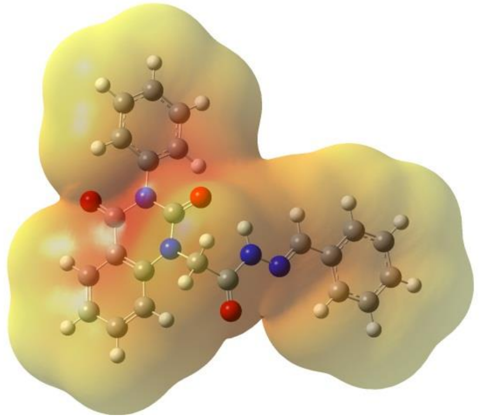

Compound 2b

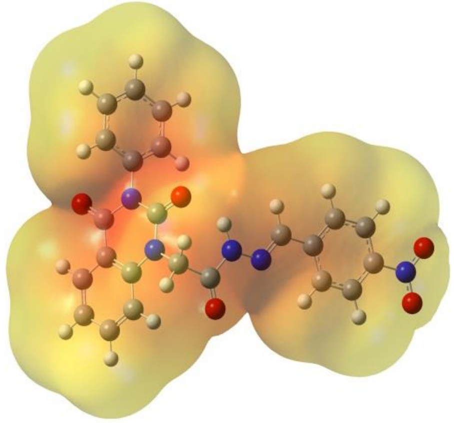

Compound 2c

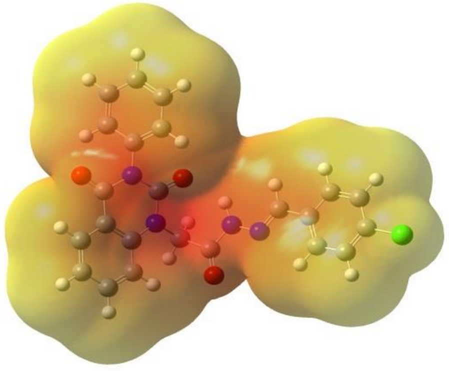

Compound 2e

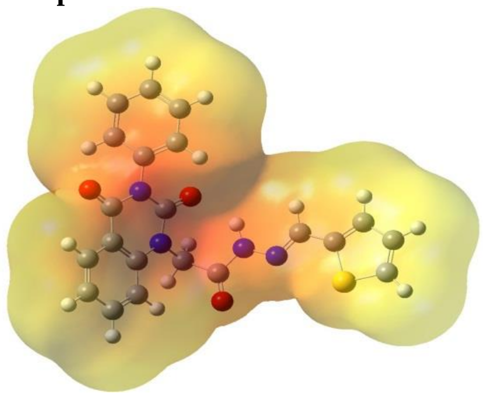

Compound 3a

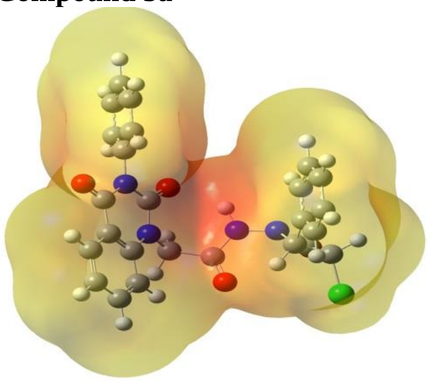

Compound 4a

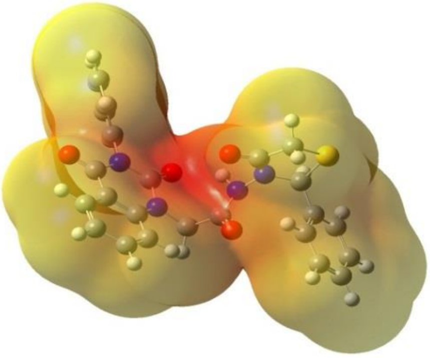

Compound 5

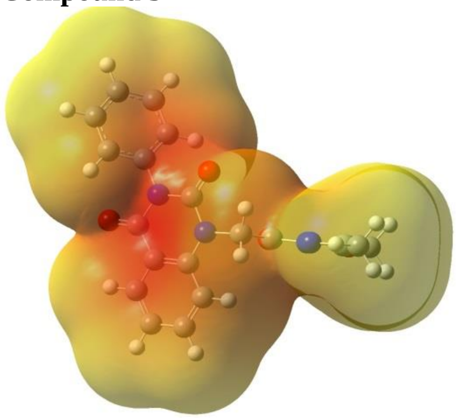

Compound 6

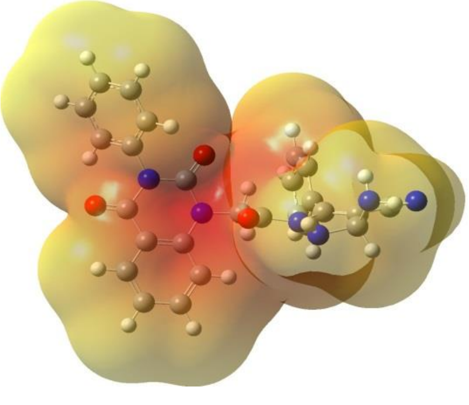

Compound 7

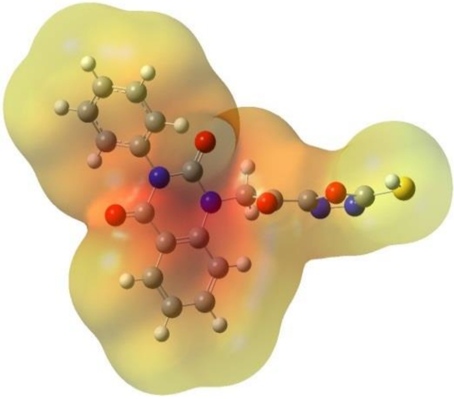

Compound 8

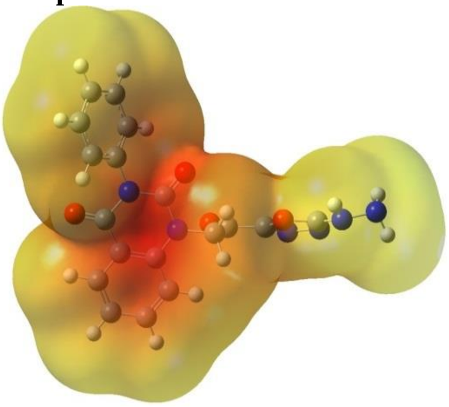

Compound 9

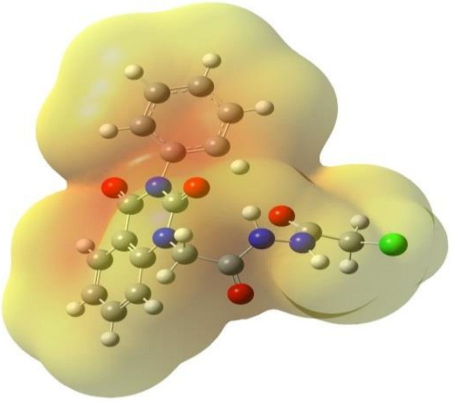

Compound 10

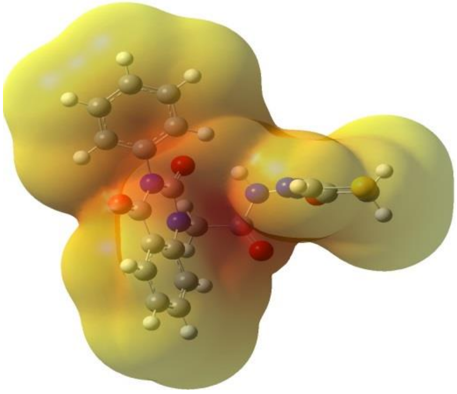

Compound 12

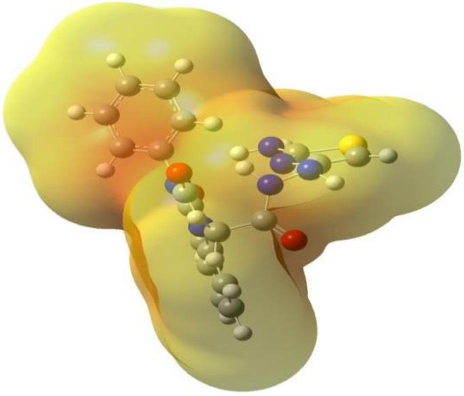

Compound 13

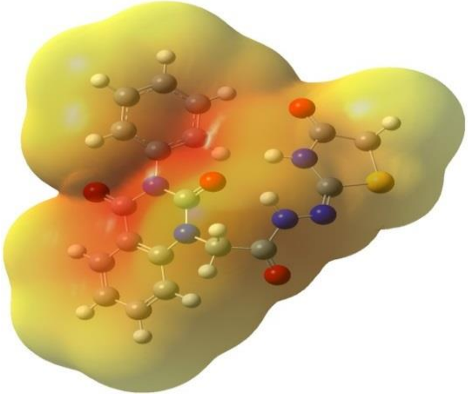

Compound 14

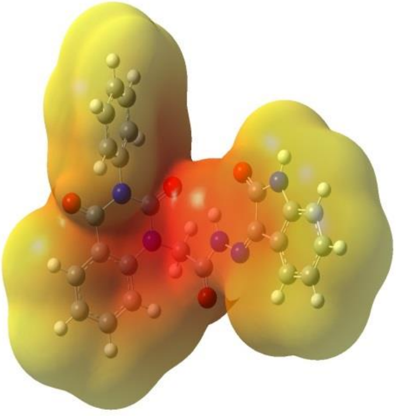

Compound 15

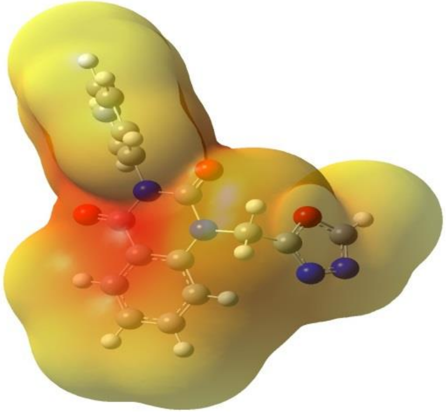

Compound 16a

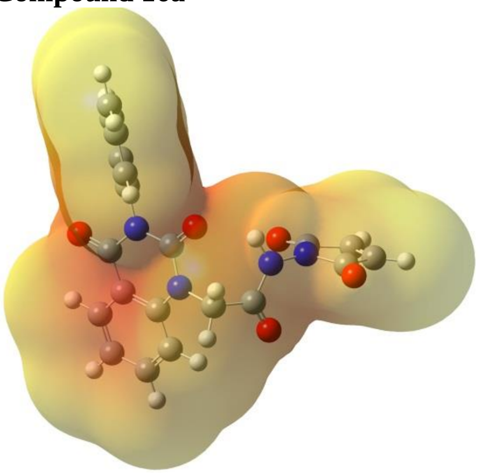

Compound 16b

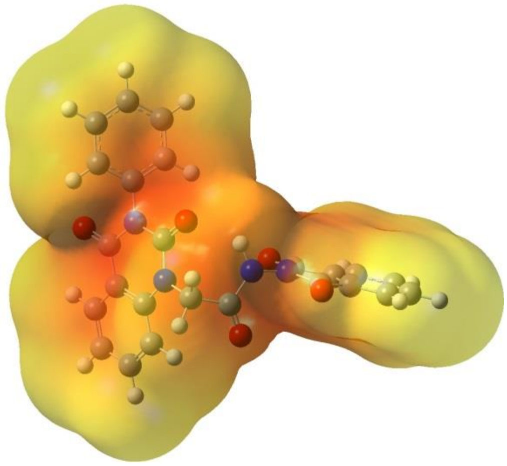

Compound 16c

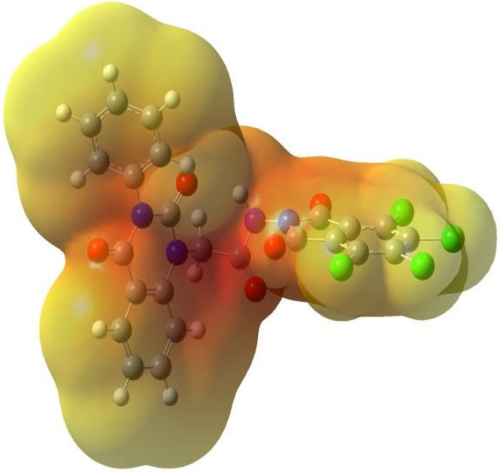

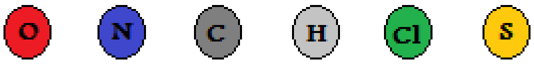

Electrostatic potential maps around the rest compounds. The red region represents highly electron density charges (electronegative), while pale yellow represents electropositive part.

Table S1. The protonation states of the amino acid residues of *pf*DHODH.

**Residue**	**pKa**
ASP204	3.97
HIS185	4.36
TYR168	11.06
TYR207	10.10
TYR528	13.80
TYR551	10.06
TYR555	12.03
LYS229	9.26
LYS260	10.74
LYS305	10.43
LYS429	8.95
ARG253	12.51
ARG262	13.68
ARG544	13.58
